# Spatial–temporal patterns and risk factors for human leptospirosis in Thailand, 2012–2018

**DOI:** 10.1038/s41598-022-09079-y

**Published:** 2022-03-24

**Authors:** Sudarat Chadsuthi, Karine Chalvet-Monfray, Suchada Geawduanglek, Phrutsamon Wongnak, Julien Cappelle

**Affiliations:** 1 Department of Physics, Faculty of Science, Naresuan University, Phitsanulok, 65000 Thailand; 2 Université de Lyon, INRAE, VetAgro Sup, UMR EPIA, 69280 Marcy l’Etoile, France; 3 Université Clermont Auvergne, INRAE, VetAgro Sup, UMR EPIA, 63122 Saint-Genès-Champanelle, France; 4 Medical and Graduate Education Division, Faculty of Science, Mahidol University, Bangkok, 10400 Thailand; 5 UMR ASTRE, CIRAD, INRAE, 34398 Montpellier, France; 6 CIRAD, UMR ASTRE, 34398 Montpellier, France

**Keywords:** Diseases, Risk factors

## Abstract

Leptospirosis is a globally important zoonotic disease. The disease is particularly important in tropical and subtropical countries. Infections in humans can be caused by exposure to infected animals or contaminated soil or water, which are suitable for *Leptospira*. To explore the cluster area, the Global Moran’s I index was calculated for incidences per 100,000 population at the province level during 2012–2018, using the monthly and annual data. The high-risk and low-risk provinces were identified using the local indicators of spatial association (LISA). The risk factors for leptospirosis were evaluated using a generalized linear mixed model (GLMM) with zero-inflation. We also added spatial and temporal correlation terms to take into account the spatial and temporal structures. The Global Moran’s I index showed significant positive values. It did not demonstrate a random distribution throughout the period of study. The high-risk provinces were almost all in the lower north-east and south parts of Thailand. For yearly reported cases, the significant risk factors from the final best-fitted model were population density, elevation, and primary rice crop arable areas. Interestingly, our study showed that leptospirosis cases were associated with large areas of rice production but were less prevalent in areas of high rice productivity. For monthly reported cases, the model using temperature range was found to be a better fit than using percentage of flooded area. The significant risk factors from the model using temperature range were temporal correlation, average soil moisture, normalized difference vegetation index, and temperature range. Temperature range, which has strongly negative correlation to percentage of flooded area was a significant risk factor for monthly data. Flood exposure controls should be used to reduce the risk of leptospirosis infection. These results could be used to develop a leptospirosis warning system to support public health organizations in Thailand.

## Introduction

Leptospirosis is a globally important zoonotic disease and causes approximately 1.03 million cases and 58,900 deaths worldwide each year^1^. The disease is particularly prevalent in tropical and subtropical climates. Infections in humans and animals can be caused by exposure to infected animals or soil and water contaminated, which are suitable for *Leptospira*^2^ and with urine from infected animals^3,4^.

Studies have found that the pathogenic *Leptospira* can survive in water and soil for months^5–7^. Climatic variables could potentially be associated with leptospirosis transmission, such as rainfall, temperature, and floods. The effects of rainfall and temperature on leptospirosis incidences were investigated in Thailand^8,9^, and at different setting and climatic zones such as in Sri Lanka^10^, China^11^, and Philippines^12^. Flooding is one of the important drivers for leptospirosis outbreaks. Leptospirosis outbreaks have been reported to be associated with flooding or flooded areas (e.g., Indonesia^13^, Pakistan^14^, Brazil^15^, and Laos^16^). As such, the risk of leptospirosis infection could be identified using remotely sensed data^11,17,18^.

In Thailand, the annual human leptospirosis cases are reported as 4.25 cases per 100,000 people, from 2012 to 2018^19^. Most reported cases are in agricultural workers, who are the most exposed to contaminated environments. A previous study in Nakhon Ratchasima province of Thailand found that rice cultivators were at risk for leptospirosis^20^. Rice paddies have been found to be associated with increased leptospirosis incidences^21,22^. A study in Nan province of Thailand found that the open habitat near rivers or in rice fields that were prone to flooding, were related to human leptospirosis infections^23^. The activities of rice farming are also associated with an increase of leptospirosis infections in other countries, for example, in Iran^24^ or Tanzania^25^. Thus far, to our knowledge, there have been few studies on the impact of rice production activities on leptospirosis infections.

Spatial–temporal variation of human leptospirosis incidences have also been investigated to identify high-risk areas, where preventive measure should be implemented^9,26–28^. The study of leptospirosis spatial–temporal patterns may help to prioritize the prevention and surveillance of leptospirosis in high-risk areas. Also, risk factors should be identified in order to control human leptospirosis.

In this work, we established a large-scale dataset of human leptospirosis in Thailand over seven years from 2012 to 2018. The objectives of this study are (1) to investigate the spatial–temporal patterns of leptospirosis incidence, (2) to determine the high-risk and low-risk provinces across Thailand, and (3) to examine the effect of rice-production-related factors, remotely sensed environmental factors, and climatic factors on leptospirosis risk in humans using a generalized linear mixed model (GLMM).

## Materials and Methods

### Data collections

Human leptospirosis case reports were retrieved from the database of national disease surveillance (report 506), Bureau of Epidemiology, Department of Disease Control, Ministry of Public Health, Thailand^19^. Most positive cases were suspected leptospirosis cases, based on the clinical diagnosis made by attending physicians and were mainly reported from public hospitals. In this research, we analysed all reported cases from 2012 to 2018 at the province level.

The percentage of flooded area was obtained from the Moderate Resolution Imaging Spectroradiometer (MODIS) of the Terra satellite (Surface Reflectance 8-Day L3 Global 500 m SIN Grid V005 (MOD09A1)). Using the surface spectral reflectance, band 4 (green) and band 7 (infrared), which measured at ground level in the absence of atmospheric scattering or absorption, the modified normalized difference water index (MNDWI) was calculated^17,29^. To calculate the percentage of flooded area, the number of pixels for which the MNDWI value was greater than or equal to zero, in each province, for each month and for each year, were counted.

To reflect the vegetation intensity, the normalized difference vegetation index (NDVI) was calculated from the value of red band (wavelength: 620–670 nm) and near-infrared (NIR) band (841–876 nm)^30^. The vegetation area was classified into two types: low vegetation (such as shrub and grassland) and high vegetation (such as temperate and tropical urban forest). The vegetation type was calculated from the percentage of NDVI pixels that had a value within 0.2–0.5 and 0.5–1, respectively^30^.

The monthly values of rainfall, minimum and maximum temperature, and soil moisture were obtained from TerraClimate^31^, calculated over a grid of approximately 4 km^2^, and aggregated at the province level. The annual average values were calculated from monthly data. We also added the temperature range (i.e., the average difference between maximum and minimum temperature) to test the model. Due to the relation of temperature range with cloud cover^32^, we hypothesize that this parameter may be used as a proxy for rainfall and flooding.

Elevation data was derived from the NASA Shuttle Radar Topographic Mission (SRTM) 90 m Digital Elevation Data^33^ as it may drive leptospirosis infection^27^. The average elevation at the province level was used in this analysis. Slope data was calculated using QGIS version 3.6.0 from the elevation data in units of degree.

The human population data was obtained from the WorldPop database (http://www.worldpop.org) and summarised as human population density per year for each province. Livestock population density (buffalo, cattle, and pigs) were obtained from the Information and Communication Technology Center (ICT), Department of Livestock Development of Thailand at the district level^34^.

In this work, we also incorporated rice production data such as rice crop arable area, rice cultivated area and rice yield, which may associate with leptospirosis occurrences. In Thailand, most cases were identified in rice farmers^35^. Rice farmers typically plant rice in two seasons, i.e., the in-season rice is called the primary rice crop and the off-season rice is called the secondary rice crop. The primary and secondary rice season data was obtained from the Office of Agricultural Economics, Thailand (http://www.oae.go.th/). The descriptions of the variables are presented in Table 1.

**Table 1:** Variables used to identify risk of infection from GLMM and their descriptions.

Variable	Description, (unit)	Reference
Temporal correlation	The current number of case reports per population of the province compared with the previous year or month	http://www.boe.moph.go.th/boedb/surdata/disease.php?ds=43
Spatial correlation	Correlation of exponential decay of distance between centroids	
Population density	Annual human population per area at each province (population/km^2^) Average human population per area at each province across 7 years (population/km^2^)	http://www.worldpop.org
Cattle density	Annual cattle population per area at each province (population/km^2^) Average cattle population per area at each province across 7 years (population/km^2^)	http://ict.dld.go.th
Pig density	Annual pig population per area at each province (population/km^2^) Average pig population per area at each province across 7 years (population/km^2^)	http://ict.dld.go.th
Buffalo density	Annual buffalo population per area at each province (population/km^2^) Average buffalo population per area at each province across 7 years (population/km^2^)	http://ict.dld.go.th
Soil moisture	Annual average of monthly soil moisture at each province Monthly soil moisture at each province, averaged across 7 years (mm)	http://www.climatologylab.org/terraclimate.html
Precipitation	Annual average of monthly precipitation at each province Monthly precipitation at each province, averaged across 7 years (mm)	http://www.climatologylab.org/terraclimate.html
Minimum temperature	Annual average of monthly minimum temperature at each province Monthly minimum temperature at each province, averaged across 7 years (°C)	http://www.climatologylab.org/terraclimate.html
Maximum temperature	Annual average of monthly maximum temperature at each province Monthly average maximum temperature at each province, averaged across 7 years (°C)	http://www.climatologylab.org/terraclimate.html
Temperature range	Difference between maximum annual average temperature and minimum annual average temperature at each province Difference between maximum monthly average temperature and minimum monthly average temperature at each province (°C)	http://www.climatologylab.org/terraclimate.html
Percentage of flooded area	The percentage of pixels with an MNDWI value greater than or equal to zero (250 m, 8 days) for each month and for each year	MOD09A1
NDVI-1	The percentage of NDVI pixels that are within 0.2–0.5 (250 m, 8 days) for each month and for each year	MOD09A1
NDVI-2	The percentage of NDVI pixels that are within 0.5–1.0 (250 m, 8 days) for each month and for each year	MOD09A1
Elevation	Average elevation of each province (90 m spatial resolution)	https://srtm.csi.cgiar.org/
Slope	Average angle of inclination at each province (90 m spatial resolution)	Calculated from elevation
Primary rice crop arable area	Annual arable area for primary rice crop at each province (km^2^)	http://www.oae.go.th/
Primary rice cultivated area	Annual cultivated area for primary rice crop at each province (km^2^)	http://www.oae.go.th/
Primary rice yield	Annual primary rice yield of each province (1000 kg/km^2^)	http://www.oae.go.th/
Secondary rice crop arable area	Annual arable area for secondary rice crop at each province (km^2^)	http://www.oae.go.th/
Second rice cultivated area	Annual cultivated area for secondary rice crop at the province level (km^2^)	http://www.oae.go.th/
Secondary rice yield	Annual secondary rice yield of each province (1000 kg/km^2^)	http://www.oae.go.th/

### Spatial–temporal autocorrelation

To determine the spatial autocorrelation, the annual incidence cases and monthly incidence cases per 100,000 population were used to calculate the Global Moran’s I index^36^ at the province level. The Moran’s I value, which ranges from − 1 to + 1, is used to classify the spatial clustering. A Moran’s I value of more than zero indicates positive autocorrelation, and a value of less than zero indicates negative autocorrelation. When the Moran’s I value is close to zero, it indicates a random distribution. The value is calculated from the spatial weight matrix, which is assigned from the nearest neighbour province. To test the significant level, we used a Monte-Carlo simulation method with 999 permutations. A significance of less than 0.05 is used to consider the cluster or no autocorrelation. We used R package ‘spdep’ to calculate the Moran’s I value^37^.

We also determined the local indicators of spatial association (LISA) at the province level. LISA was used to describe leptospirosis clusters with hot spots (High–High), cold spots (Low–Low) and spatial outliers (High–Low and Low–High)^38^. Hot spots or High-High provinces indicate provinces that had a high leptospirosis incidence rate and were surrounded by provinces with high incidence rates. Cold spots represent provinces with low incidences surrounded by other low incidence provinces. Spatial outliers represent high incidence provinces surrounded by low incidence provinces (High–Low) and low incidence provinces surrounded by high incidence provinces (Low–High). LISA was calculated using GeoDA version 1.18 software (https://geodacenter.github.io/)^39^. The spatial weights file was created based on an inverse distance function with a distance band of 150 km. We considered a p-value less than 0.05 as a statistically significant result using 999 permutations.

### Statistical model

To study the spatial and temporal distribution of leptospirosis, we used a generalized linear mixed model (GLMM). Mixed effect models were used to account for the spatial dependence using province ID and for the temporal dependence, using time as random effect variables. The response variable of this model was the number of reported cases. Due to the data containing many zeros, the model would underpredict the cases. In this work, we used a zero-inflated negative binomial distribution (ZINB), which is an extension of the Poisson regression and captures the excess number of zeros^40,41^. To fit ZINB, we used the R package ‘glmmTMB’^42^. We also added spatial and temporal correlation terms to consider the spatial and temporal structures (Table 1).

All variables were scaled by subtracting their mean and dividing by the standard deviation before investigating the effect of factors. We first selected the variables by studying the cross-correlation. For pairs of variables that have highly correlated, (more than |0.5|), only one variable was selected based on the minimum AIC value of the univariate model. Then, multivariate models, including all selected variables, were examined using the dredge function from the R package ‘MuMIn’^43^. This function generates the 2^N^ model table with subsets of fixed effect terms, where N is the number of variables. The best models are sorted based on the criterion (AIC). The final model was the model with the least number of variables in the set of models with a ΔAIC < 2 in comparison with the model of the lowest AIC.

### Ethics statement

All information of leptospirosis surveillance data were collected from the Thai Ministry of Public Health. This study was approved by the Institutional Review Board of Naresuan University (P10003/64). The need of informed consent was waived by the Institutional Review Board of Naresuan University as all data of our study are deidentified. The analyses were performed at aggregate level (province level) and no confidential information was involved. All methods were performed in accordance with the relevant guidelines and regulations. The location and date of onset of illness were also gathered. The maps presented in this paper do not identify patients’ addresses.

### Software

For the maps and for data analysis, we used R program version 4.1.0^44^ with package tidyverse 1.3.0^45^, stringr 1.4.0^46^, spdep 1.1–5^47^, sf 0.9–6^48^, and ggpubr 0.4.0^49^. The cross-correlation plots were created using R package ggcorrplot 0.1.3^50^. For the heat map created using Microsoft Excel version 16.57 (https://www.microsoft.com/th-th/microsoft-365/excel).

## Results

### Descriptive results

**Figure 1:**
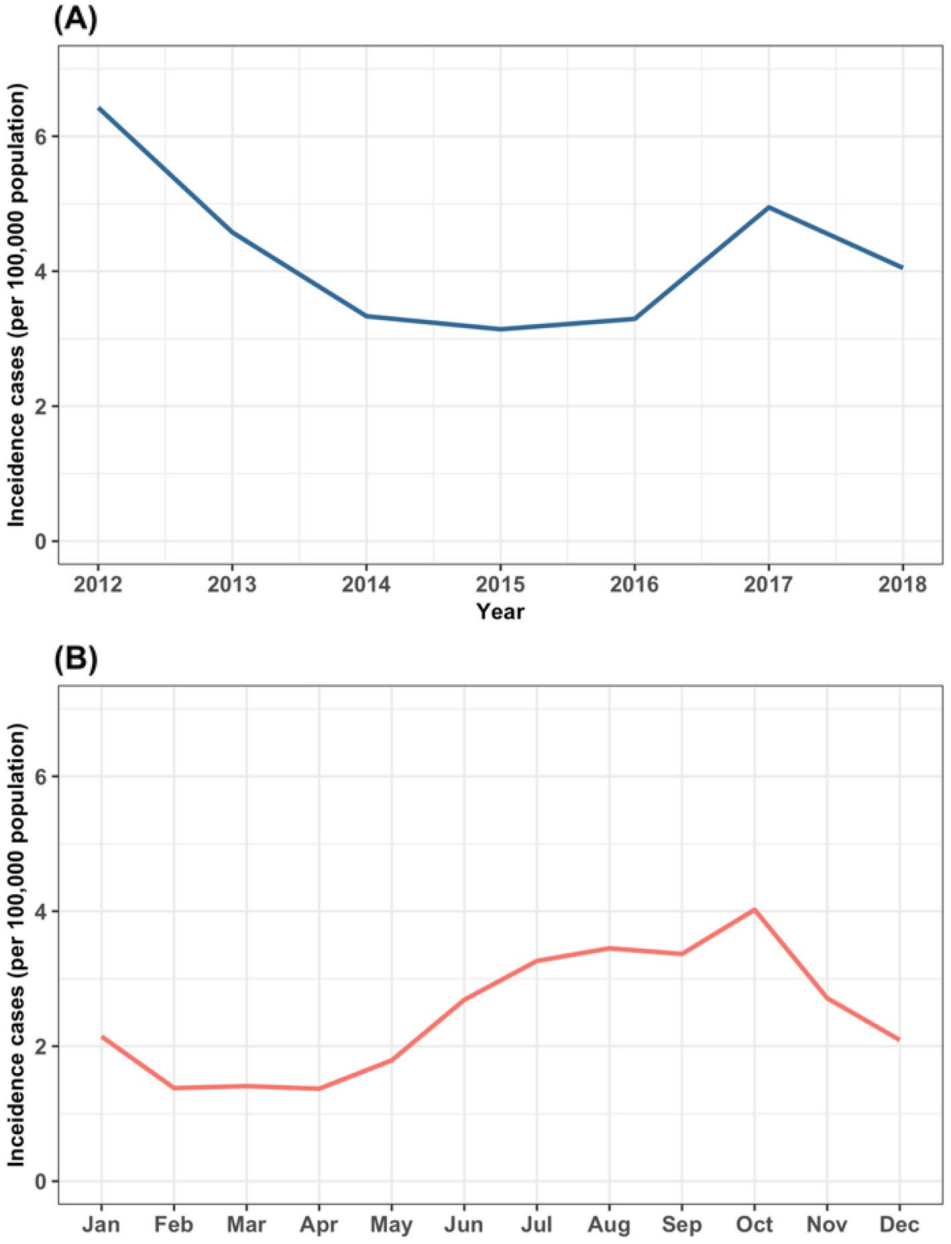
Annual (**A**) and monthly (**B**) leptospirosis incidence rates in Thailand (per 100,000 population).

From 2012 to 2018, there were 20,459 reported leptospirosis cases in Thailand. The annual incidence per 100,000 population (incidence rate) is plotted in Fig. 1. The incidence rate ranged from 3.1 in 2015 to 6.43 in 2012. After 2012, the incidence rate declined till 2014, where it levelled off, then slightly increased after 2016. In addition, the monthly incidence rate was also analysed (Fig. 1). We found that the incidence rates were higher, than the average of 2.47, between June and November, with the highest value in October. Spatial variation was observed in the northern and southern parts Thailand, while the incidence rate in the north-eastern part was observed all year round (Figs. S1 and S2). The incidence rate in the central part was found to be lower than other regions.

### Spatial–temporal analysis

**Figure 2:**
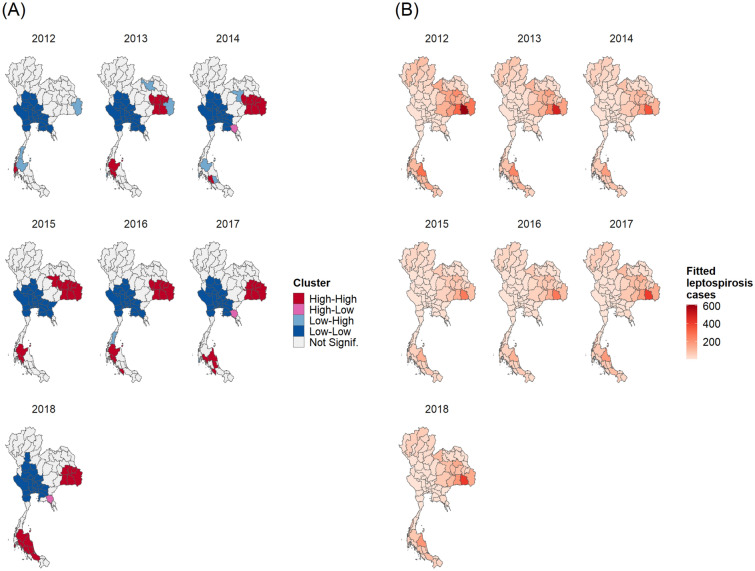
The plots of annual spatial clustering of incidence rate as determined by LISA (**A**) and annual leptospirosis cases estimated by the final GLMM (**B**). Maps created using R Program version 4.0.3 (https://www.r-project.org/).

**Figure 3:**
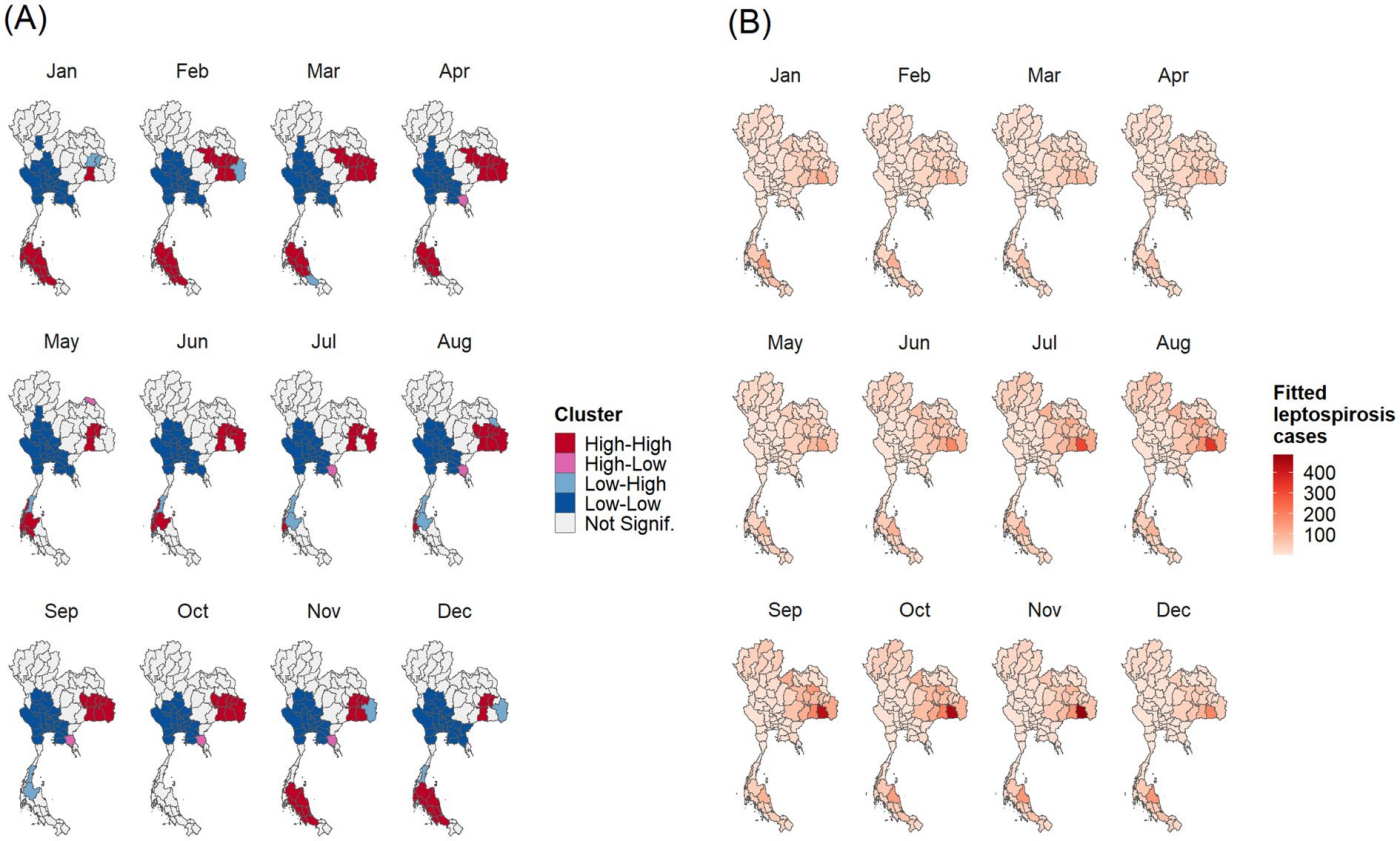
Plots of monthly spatial clustering of incidence rate as determined by LISA (**A**) and monthly leptospirosis cases estimated by the final GLMM (**B**). Maps created using R Program version 4.0.3 (https://www.r-project.org/).

**Figure 4:**
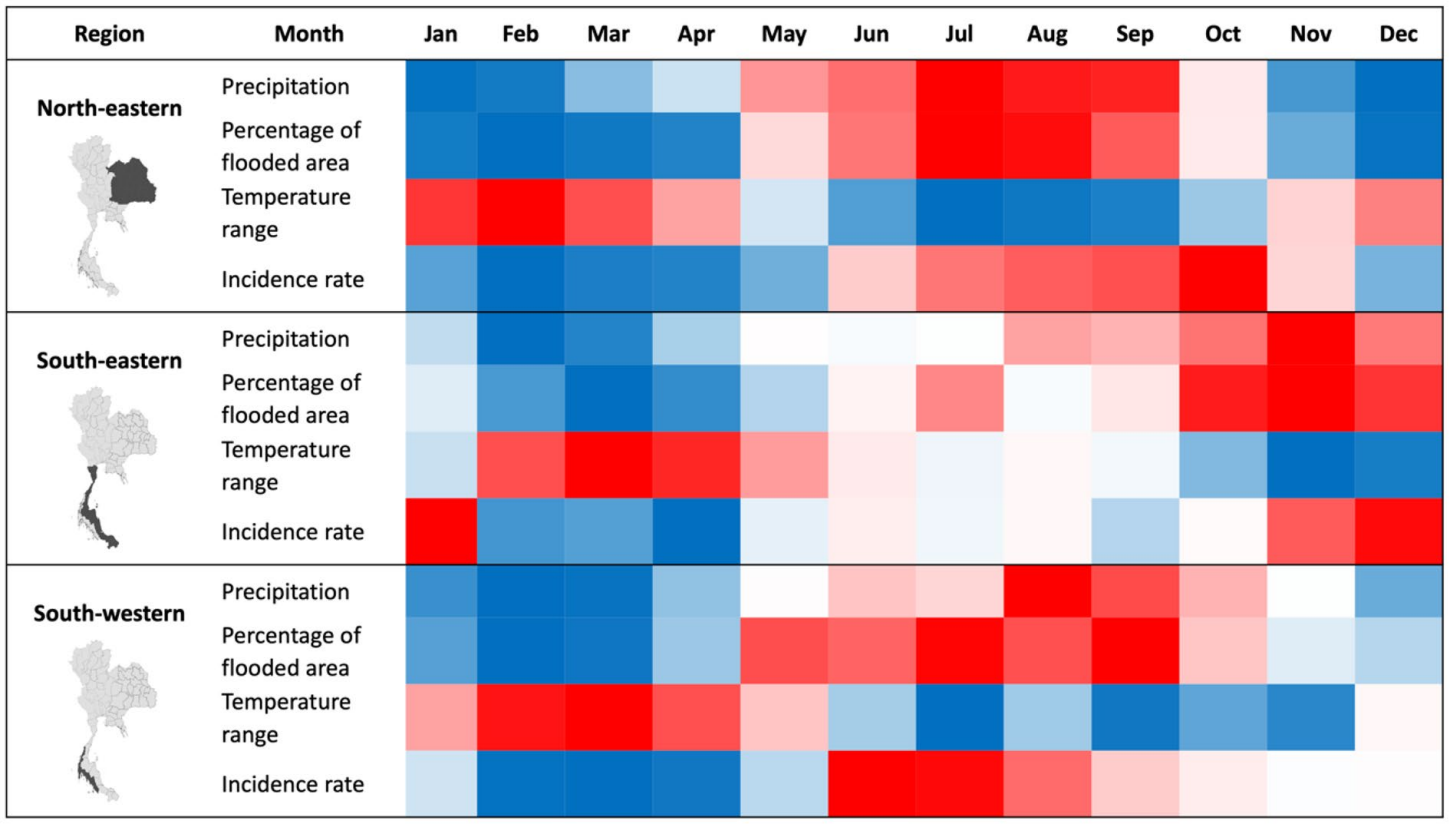
Heatmaps of monthly precipitation, monthly percentage of flooded area, monthly temperature range, and average of incidence rate for 3 regions. Red highlights the high values. Blue highlights the low value. The 3 regions were mapped in dark grey. The heat map created using Microsoft Excel version 16.57 (https://www.microsoft.com/th-th/microsoft-365/excel). Maps created using R Program version 4.0.3 (https://www.r-project.org/).

To investigate the spatial autocorrelation, the Global Moran’s I values were calculated (Tables S1 and S2). The results showed significant positive values, demonstrating a non-random distribution throughout the period studied, for both annual and monthly incidence rates. To identify high-risk provinces, results of the analysis of local indicators of spatial association were mapped in Figs. 2A and 3A. For the annual data, the LISA maps show that high-high clusters appeared in the lower north-east, except in 2012, the year with the lowest Moran’s I value. In the south, the high-risk provinces display different patterns. For monthly data, we found high-risk provinces in north-east during rainy season correspond to Fig. 4. The high-risk areas were observed in the south-west correspond to high rainfall (Fig. 4). However, for south-east, the high-risk areas were observed all months except in September and October. Low-risk provinces were mainly detected in central Thailand for both annual and monthly data. Whereas the LISA results of the north showed that there were no significant clusters.

### Risk factors

To select explanatory variables in the model for annual reported data, we calculated the cross-correlation of the scaled annual variables (Fig. S3). Because precipitation, percentage of flooded area, temperature range, and elevation were highly correlated, they could not be selected in the same model. Elevation, the variable with the lowest univariate model AIC (best model) was kept for model selection. The set of best models (with a ΔAIC < 2 to the model with the lowest AIC) is shown in Table S3 and, following the parsimony principle, we selected the model with the lowest number of variables within this set of best models. Thus, for annual reported data, the final model included annual population density, elevation, and primary rice crop arable areas. These three variables were positively associated with the annual reported cases (Table 2). The set of these explanatory variables is mapped and shown in the supplementary data (Figs. S7–S15).

**Table 2:** Results of the final generalized linear mixed model for annual reported cases.

Variables	OR (95% confidence interval)	P-value
Primary rice crop arable area	1.9576 (1.4398–2.6617)	< 0.0001
Elevation	1.9259 (1.3125–2.8259)	0.0008
Annual population density	1.2708 (1.0213–1.5812)	0.0316

*OR* odds ratio.

**Figure 5:**
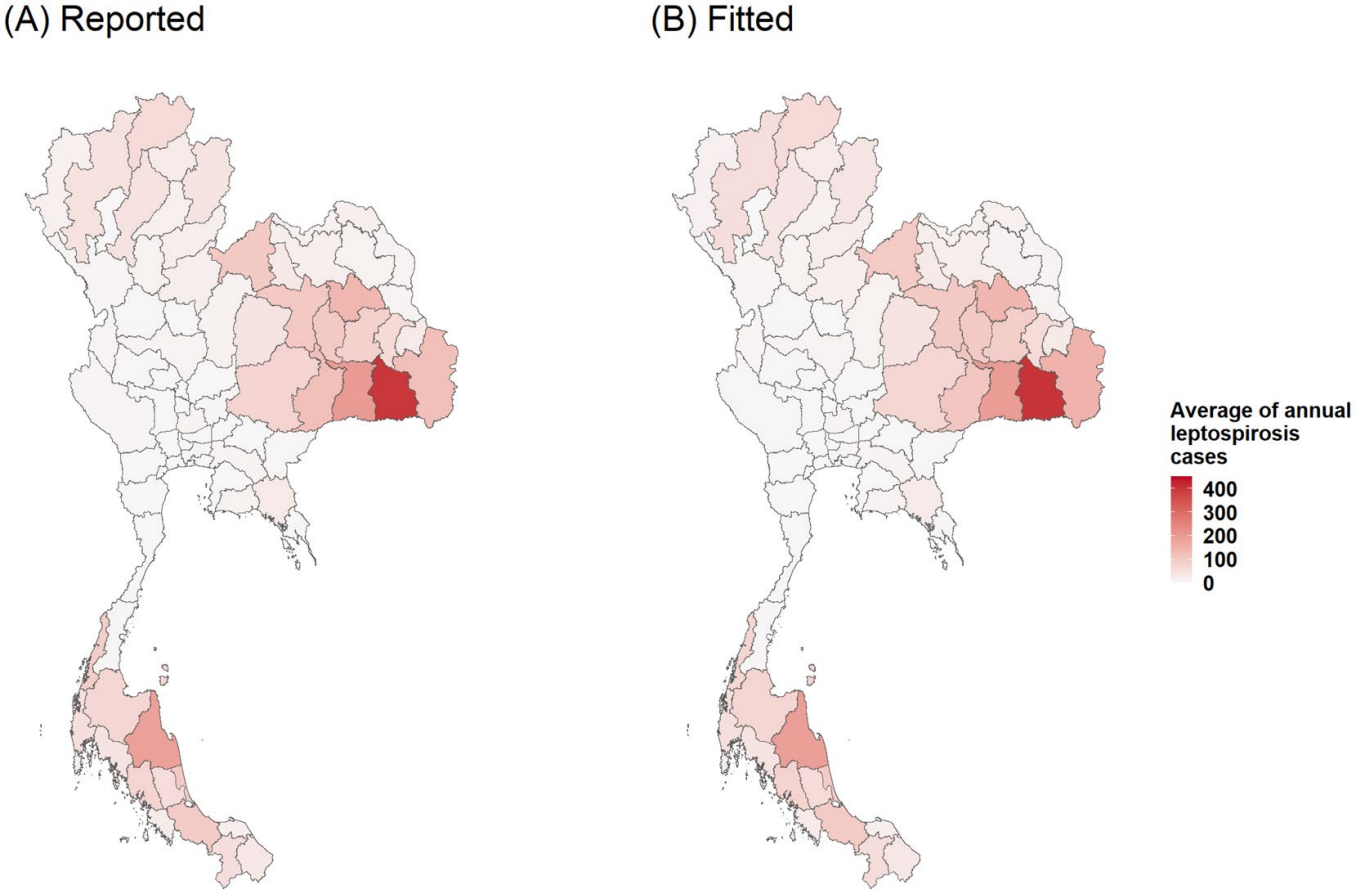
Plots of comparison between average annual reported leptospirosis cases (**A**) and average annual fitted leptospirosis cases estimated by the final GLMM (**B**). Maps created using R Program version 4.0.3 (https://www.r-project.org/).

Higher population density was generally found to increase the risk of infection. Elevation was also a predictor for leptospirosis. Specifically in the north-east region, we found that the primary rice crop arable areas were associated with a high probability of leptospirosis (Fig. S13). The estimated annual leptospirosis cases in each year are mapped in Fig. 2B. We found high numbers of predicted cases of leptospirosis in all parts of Thailand except for the Central region, where there were relatively few cases. Figure 5 showed that our model can fit to the reported cases. Note that the weather variables were not included in the final model because of the selection criteria.

The same procedure was applied to the monthly data. For monthly reported data, the variables for selecting the final model were temporal correlation, pig density, buffalo density, monthly average soil moisture, monthly temperature range, and monthly NDVI-2 percentage (Table S4). The set of selected variables is also mapped in the supplementary data (Figs. S15–S21). The set of models with a ΔAIC < 2 is shown in Tables S4 and S5. We found that the model using temperature range showed a lower AIC compared to the one using the percentage of flooded area. However, the model using flooded area used less variables, as NDVI-2 was not included. The results from both final models are presented in Table 3 and Table S6. For both models, the temporal autocorrelation parameter was included, indicating the positive correlation of the previous month. The monthly average soil moisture was found to be a positive correlation to the cases, implying the greater the soil moisture the more risk of infection. Temperature range was found to be strongly negative correlation to percentage of flooded area (r ≤ − 0.7). Also, the monthly NDVI-2 percentage were negatively with the monthly reported cases. With the alternative final models (Table S6), we found that the percentage of flooded area is positively correlated with monthly reported cases.

**Table 3:** Results of the final generalized linear mixed model for monthly reported cases.

Variables	OR (95% confidence interval)	P-value
Monthly temporal correlation	1.1253 (1.0843–1.1678)	< 0.0001
Monthly soil moisture	1.3206 (1.2257–1.4230)	< 0.0001
Monthly temperature range	0.7869 (0.7462–0.8297)	< 0.0001
Monthly percentage of NDVI-2	0.9533 (0.9186–0.9893)	0.0115

*OR* odds ratio.

**Figure 6:**
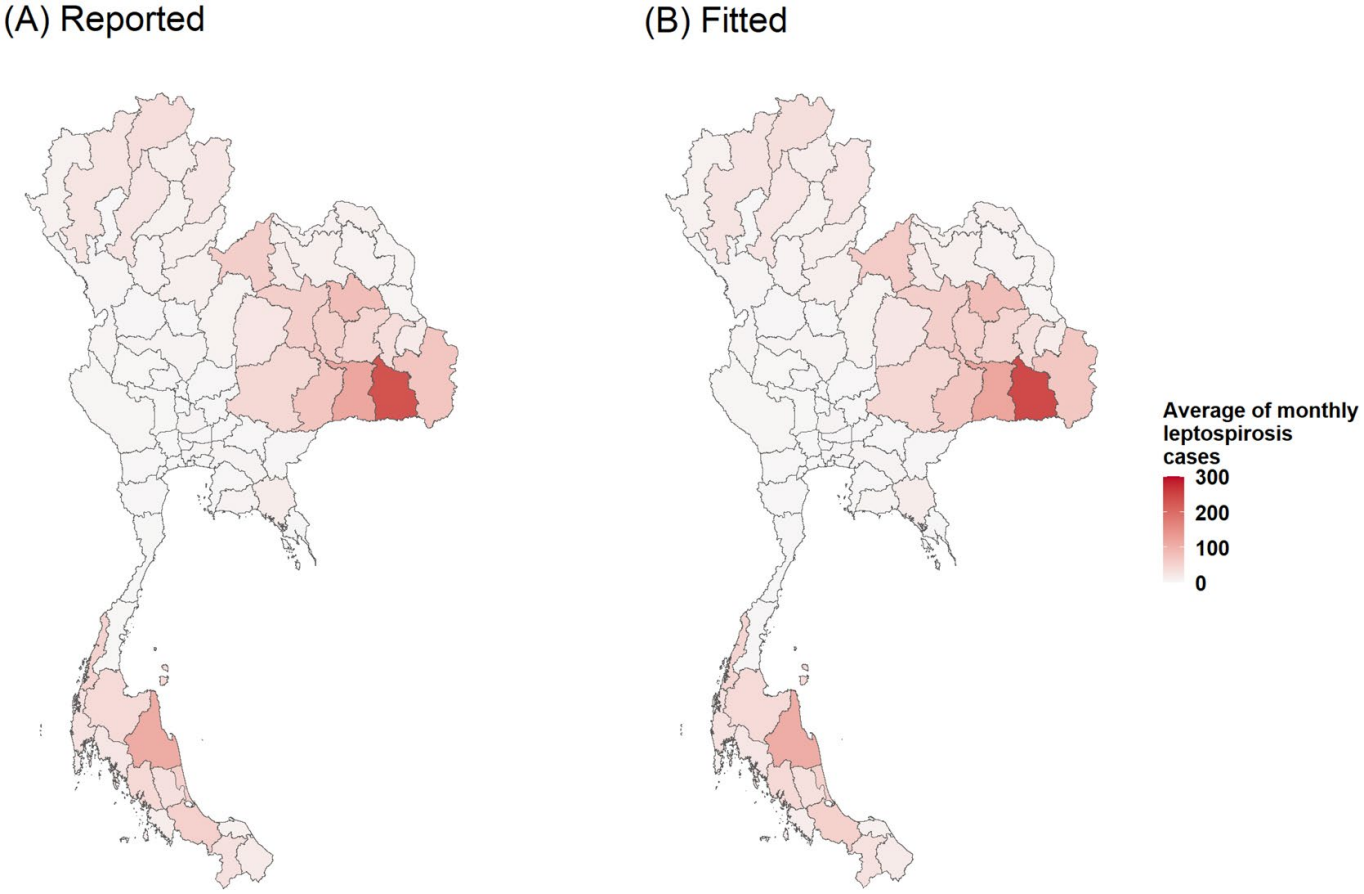
Plots of comparison between average monthly reported leptospirosis cases (**A**) and average monthly fitted leptospirosis cases estimated by the final GLMM for temperature range parameter (**B**). Maps created using R Program version 4.0.3 (https://www.r-project.org/).

The results indicated that low temperatures together with a high percentage of flooded area could increase infection risk. The estimated monthly leptospirosis cases are mapped in Fig. 3B. Both models can be used to illustrate the average monthly leptospirosis cases as shown in Fig. 6, Figs. S5, S6.

## Discussion

The spatial–temporal analysis of all human leptospirosis cases reported in Thailand from 2012 to 2018 allowed us to highlight the main spatial and temporal patterns and to identify key risk factors associated with leptospirosis infection in Thailand. There are few studies of the spatial–temporal patterns of leptospirosis in the country and none to our knowledge using such a large data set. Although the association of climatic and environmental factors on leptospirosis has been studied in many previous works^51,52^, few studies used remotely sensed data to analyze directly the impact of flooding and took into account primary and secondary rice crop arable land, cultivated areas and yields.

Overall, our analysis showed high incidence rates for specific provinces and seasons in Thailand, suggesting that leptospirosis is still a major public health concern as it included in the prevention research program to control disease by Department of Disease Control, Ministry of Public Health. The spatial autocorrelation analysis highlighted the significance of the annual and monthly spatial clustering of the leptospirosis cases. For the annual data, there are limited differences between the years, as the same provinces (provinces from north-eastern Thailand) have higher risk almost every year. Whereas for the monthly data, the high-risk provinces were different between the months for different parts of the country, especially for the north-eastern and southern parts. It is interesting that in October, which has the highest incidence rate, the high-risk provinces were observed only in north-eastern parts. This may be explained by the process of rice cultivation, which involves many activities such as preparing the land, sowing the wet fields and weeding as discussed below. The hot spots in the north-eastern are almost exclusively rural areas. On the other hand, the hot spots in the south may not be related only to rice cultivation but rather to flooding, as an outbreak occurred 2 weeks after flooding in Nakhon Si Thammarat in January 2017^53^. It also may have a different seasonality component as compared to the north-eastern part^54^ and a different landscape. The low-risk provinces were clustered in the central region of Thailand, which has the highest rice productivity in the country^55^.

Our analysis highlights that the areas of different rice cultivated types (in-season and off-season) and rice yield should be considered separately to describe the risk of leptospirosis infection. Consistent with our results, rice farming activities have been identified to be an important risk factor^20,24,25^. In Thailand, rice farming is the predominant occupation and rice farmers usually cultivate rice in two seasons. Our results demonstrated that the primary rice crop arable area (rice grown using rainwater) was positively associated with the annual leptospirosis incidence. The primary rice crop (in-season) is cultivated during the rainy season (May–October), thus increasing activities in the field, such as fertilizing and ploughing rice in wet fields, leading to a higher risk of exposure to *Leptospira*. In contrast, the secondary rice crop arable area (off-season) was not considered be a risk factor for leptospirosis as it corresponded to rice growing activities during the non-rainy season. However, rice yield, mainly in the central region, was found to be negatively correlated to primary rice crop arable area. The central region has soil conditions more suitable for increased rice production per area and higher water resources allowing for several rice harvest per year. Our results may suggest that the contamination of *Leptospira* may depend on the soil characteristics^56^. It may also be due to the different practices for rice cultivation in the central region, which has more mechanized processes than farmers in north-eastern region. Another reason may be the highly effective healthcare system in the central region compared to other parts of the country. The leptospirosis infection could be explained by using land use such as rice growing data and landscape such as elevation. Elevation may be a proxy for increased flooding because lower elevations store rainwater. However, elevation was calculated at province level, the finer spatial resolution should further analysis to be more specific calculation. Due to the limitation of the data (annual data for rice yield), further analysis for the monthly data should be evaluated to calculate the risk factors associated with different seasons.

For the monthly data, we found that using temperature data leads to a minor improvement in the model compared to using the percentage of flooded area. The results showed both parameters can be predictor variables for leptospirosis. Our analyses revealed that temperature has a strongly negative correlation to percentage of flooded area. Usually, in tropical climates, low temperatures correlate to cloudy weather, where there is high rainfall and therefore prone to flood^32^. The temperature range could be a predictor variable, when the percentage of flooded area is not available. However, using temperature range instead of the percentage of flooding area should be only used in similar climatic areas as Thailand where the model was built and where the correlation between these two factors is likely to hold true. More generally, any extrapolation of this model to other climatic regions should be implemented carefully.

Our finding allows the potential risk of leptospirosis infection to be estimated almost in real-time. *Leptospira* can survive in freshwater^2^ where flooding events could increase the number of pathogenic *Leptospira*^57^. The average soil moisture, or the soil water capacity^31^, was identified as a risk factor for monthly data in this work. *Leptospira* can survive in soils with a moisture content of ≥ 20%^58^. Increased soil moisture may increase the survival rate in contaminated soil and water^26^. Note that our results were based on the case reports in the surveillance system. This may not be accurate because some mild symptoms or asymptomatic cases are not going to the hospital, resulting in underreporting of leptospirosis cases. A finer spatial and temporal scale should also be conducted, when well-represented data is available. The analysis could only be performed using the data set by the month and year due to the low number of cases. Regardless of these limitations, our study has provided important knowledge on leptospirosis occurrences by characterizing the hot-spots and key risk factors.

## Conclusion

In summary, our study highlighted that when studied over a long period, leptospirosis cases demonstrated a spatial–temporal distribution and showed hot-spots and clustered areas. Regression analysis was used to find the risk factors associated with leptospirosis cases. Working in rice field, which have high soil moisture, could increase risk of infection due to occupational behaviour of working in wet fields facilitating the survival of *Leptospira* bacteria. The temperature range, which is negatively correlated to the percentage of flooded area was found to be negatively associated with cases. Therefore, flood exposure controls should be used to reduce the risk of leptospirosis infection. These results could be used to improve prevention measures and control actions for public health organizations. The investigation of the associated risk factors can contribute to the prevention and early warning of leptospirosis in Thailand.

## Supplementary Information

Supplementary Information.

## Data Availability

The leptospirosis dataset used in the current study are available from Bureau of Epidemiology, Department of Disease Control, Ministry of Public Health, Thailand upon reasonable request. The data supporting the findings can be found in the main paper and in Supplementary Information file.
