# Supplementary material for: Spatial–temporal patterns and risk factors for human leptospirosis in Thailand, 2012–2018
Source: Sci Rep. 2022 Mar 24;12:5066. doi: 10.1038/s41598-022-09079-y (PMC8948194; doi:10.1038/s41598-022-09079-y)
Supplement: Supplementary file 1 — Supplementary Information. [file 41598_2022_9079_MOESM1_ESM.docx]

**Supplementary file**

**Spatial-temporal patterns and risk factors for human leptospirosis in Thailand, 2012-2018**

Sudarat Chadsuthi^1*^, Karine Chalvet-Monfray^2,3^, Suchada Geawduanglek^4^, Phrutsamon Wongnak^2,3^, Julien Cappelle^2,5,6^

^1^Department of Physics, Faculty of Science, Naresuan University, Phitsanulok 65000, Thailand

^2^Université de Lyon, INRAE, VetAgro Sup, UMR EPIA, 69280 Marcy l’Etoile, France

^3^Université Clermont Auvergne, INRAE, VetAgro Sup, UMR EPIA, 63122 Saint Genès Champanelle, France

^4^Medical and Graduate Education Division, Faculty of Science, Mahidol University, Bangkok 10400 Thailand

^5^UMR ASTRE, CIRAD, INRAE, 34398, Montpellier, France

^6^CIRAD, UMR ASTRE, F-34398, Montpellier, France

**Corresponding author:** Sudarat Chadsuthi

Department of Physics, Faculty of Science, Naresuan University, Phitsanulok 65000, Thailand

E-mail : sudaratc@nu.ac.th

**1. Supplementary Results**


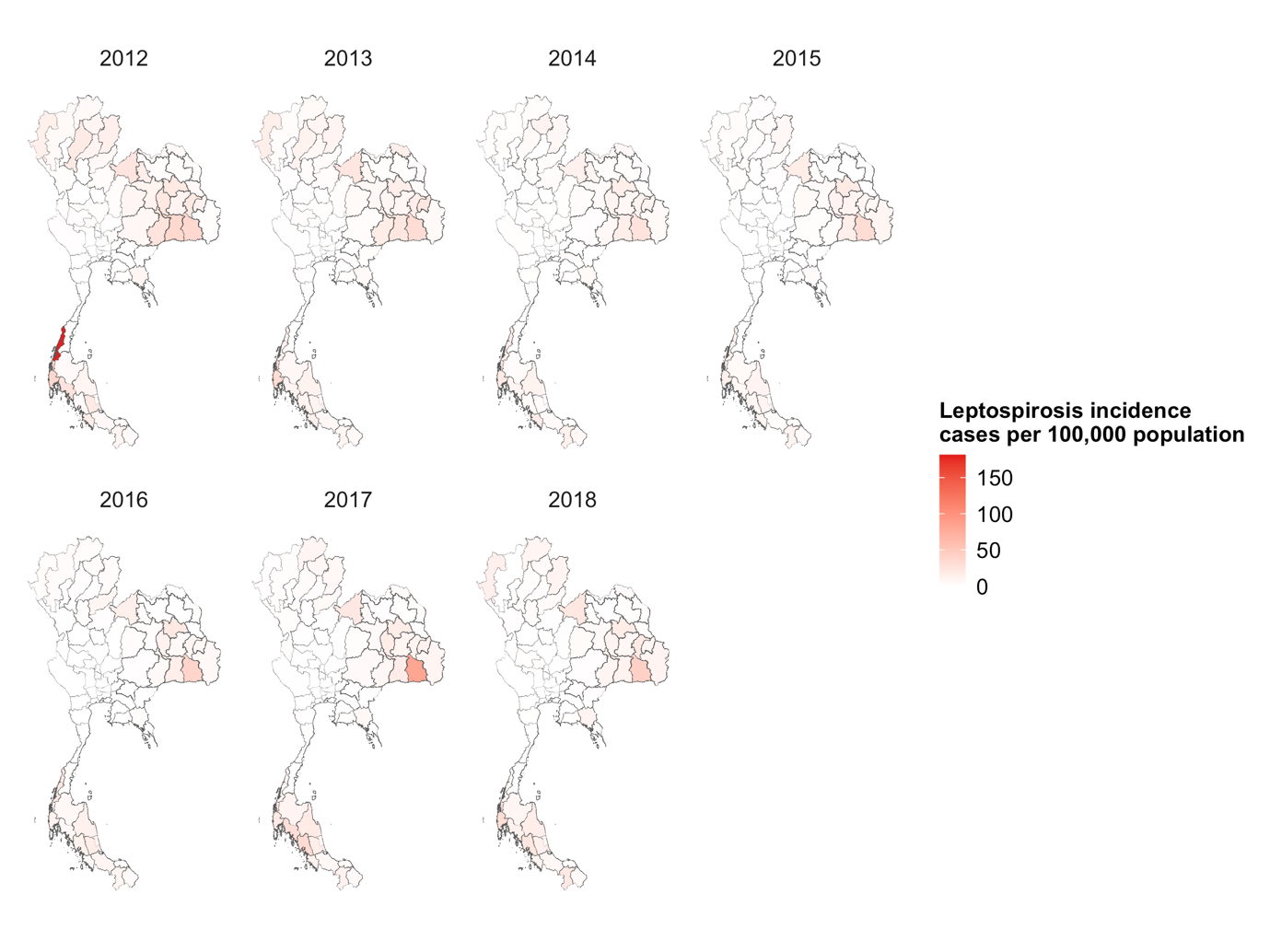


**Fig. S1.** The spatial-temporal pattern of annual leptospirosis incidence rate (per 100,000 population) cases during 2012–2018. Maps created using R Program version 4.0.3 (https://www.r-project.org/).


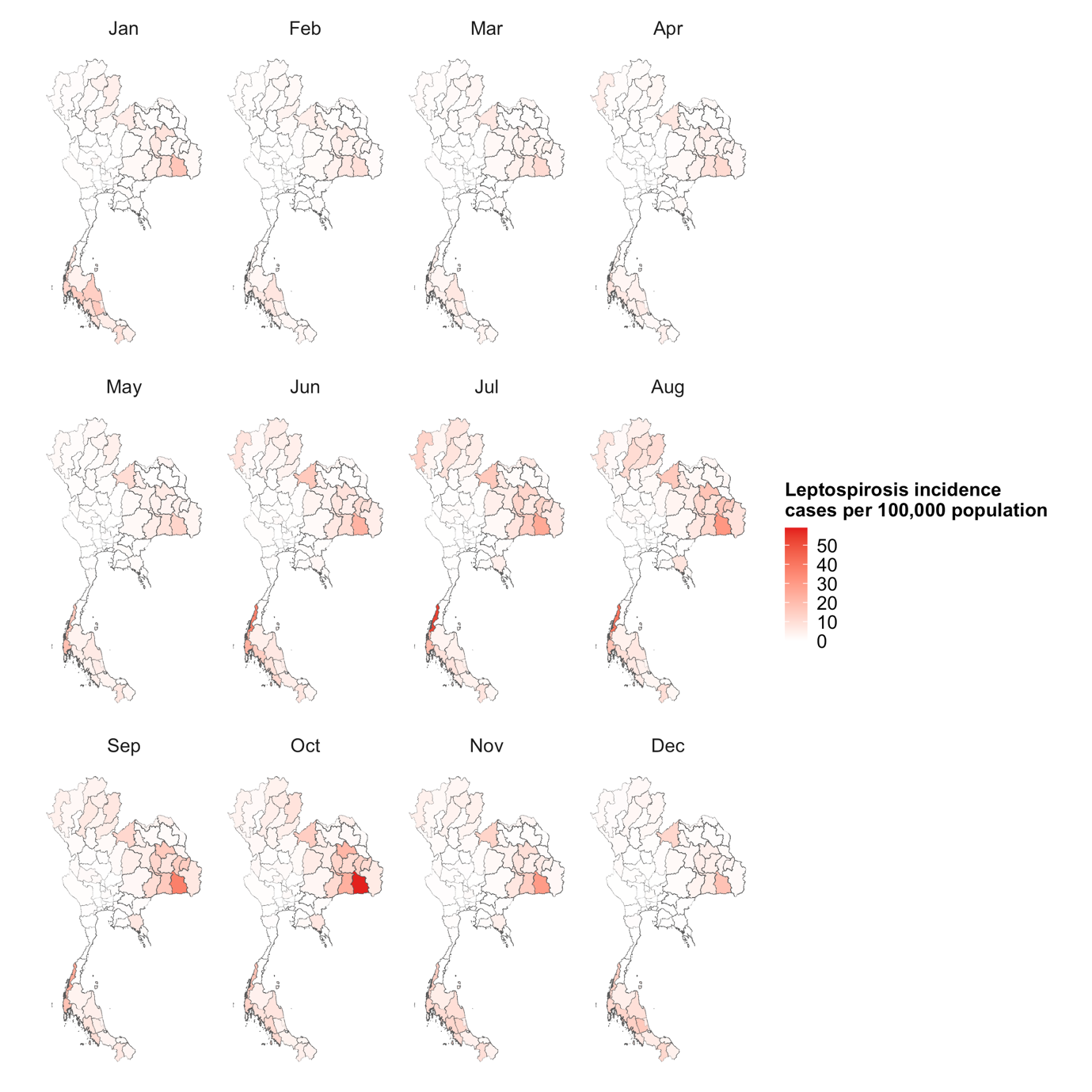


**Fig. S2.** The spatial-temporal pattern of monthly leptospirosis incidence rate (per 100,000 population) cases during 2012–2018. Maps created using R Program version 4.0.3 (https://www.r-project.org/).


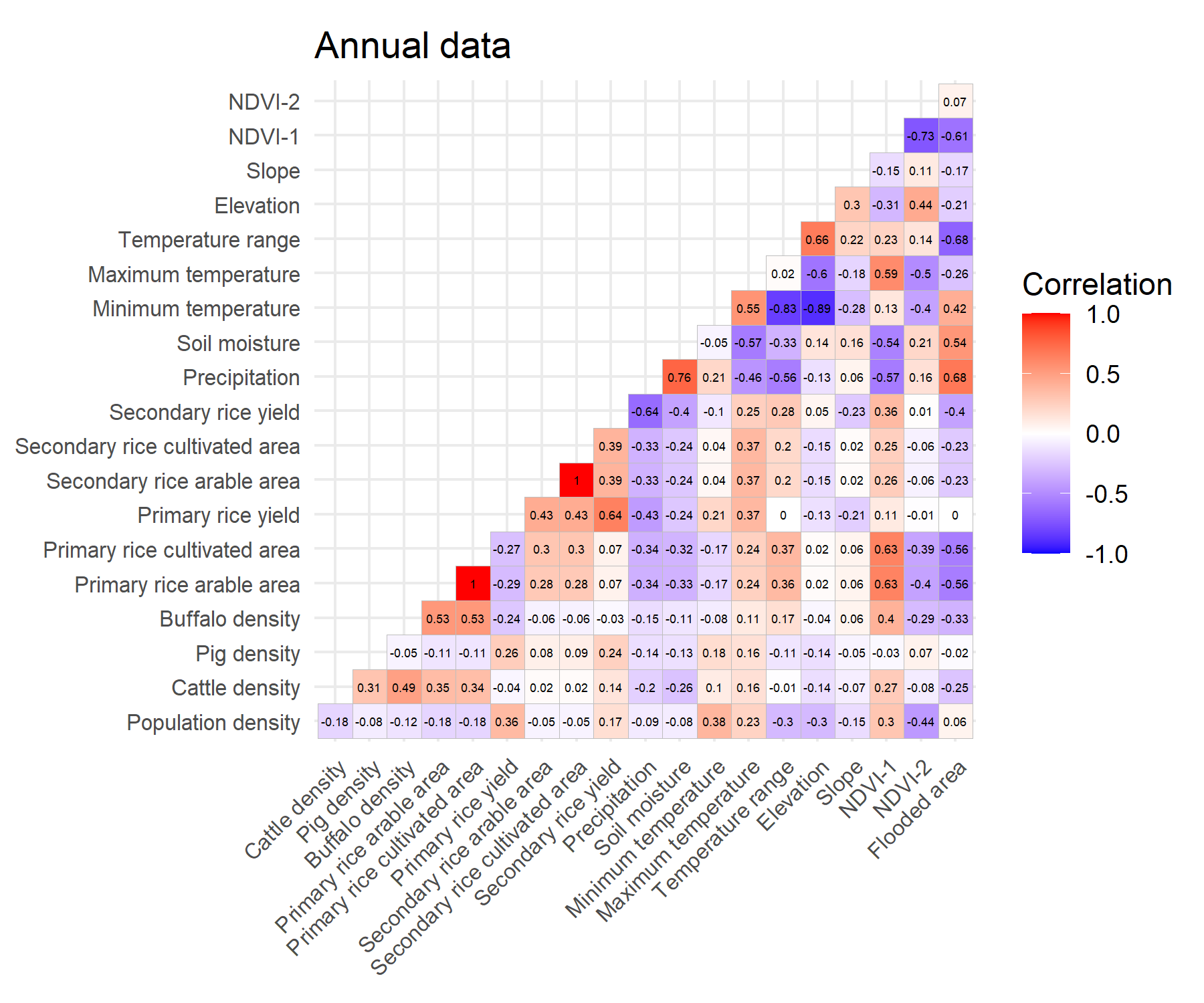


**Fig. S3.** Cross**-**correlation of the annual variables. The plot created using R Program version 4.0.3 (https://www.r-project.org/).


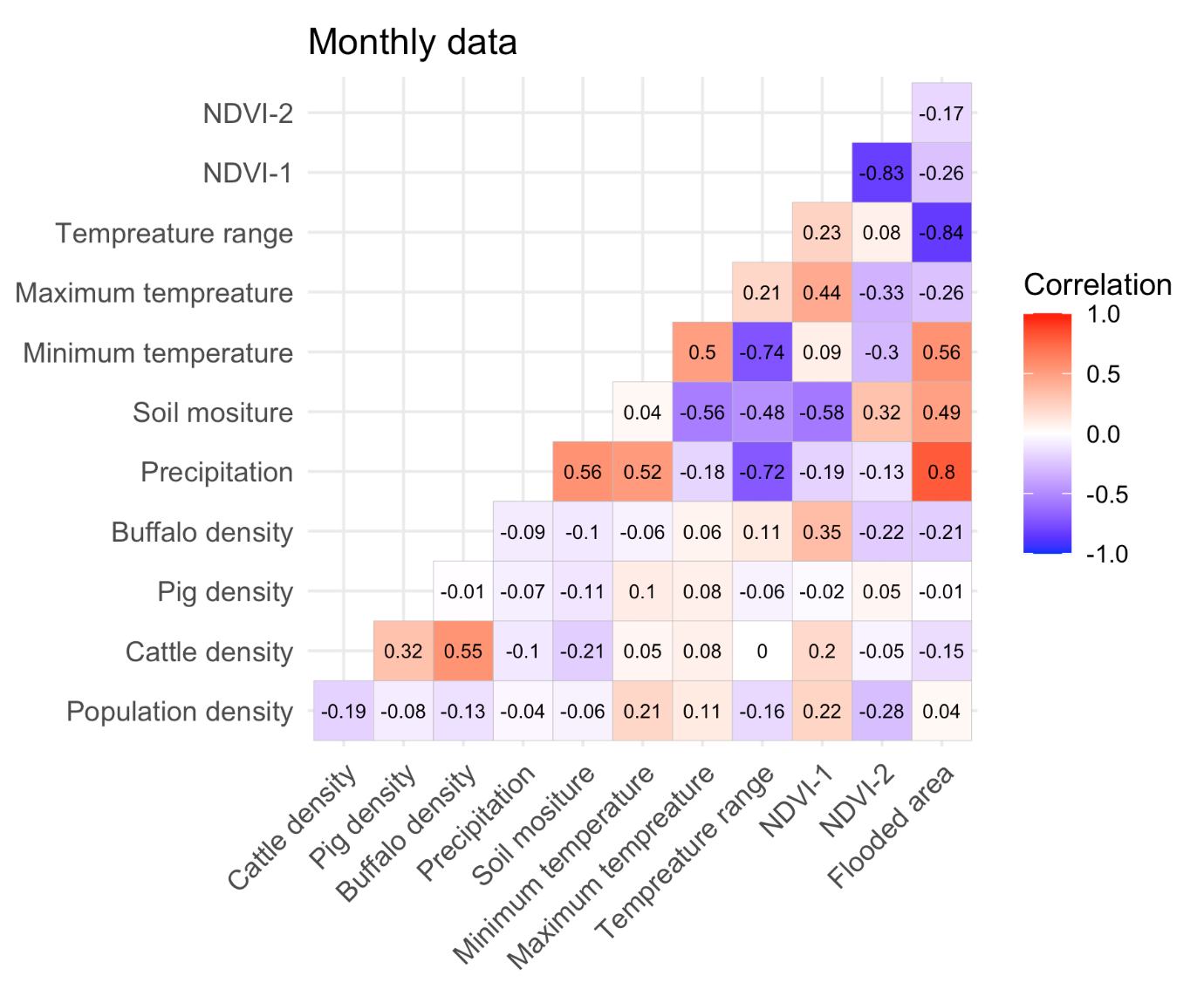


**Fig. S4.** Cross**-**correlation of the monthly variables. The plot created using R Program version 4.0.3 (https://www.r-project.org/).

**Table S1.** Global Moran’s I of annual leptospirosis incidence rate in Thailand from 2012–2018

| Year | Moran’s I | P-value |
| --- | --- | --- |
| 2012 | 0.1238 | 0.004 |
| 2013 | 0.3362 | 0.001 |
| 2014 | 0.3605 | 0.001 |
| 2015 | 0.4240 | 0.001 |
| 2016 | 0.3521 | 0.001 |
| 2017 | 0.3153 | 0.001 |
| 2018 | 0.3758 | 0.001 |

**Table S2.** Global Moran’s I of monthly leptospirosis incidence rate in Thailand

| Month | Moran’s I | P-value |
| --- | --- | --- |
| January | 0.5567 | 0.001 |
| February | 0.5495 | 0.001 |
| March | 0.4529 | 0.001 |
| April | 0.4295 | 0.001 |
| May | 0.3993 | 0.001 |
| June | 0.3695 | 0.001 |
| July | 0.2562 | 0.003 |
| August | 0.3156 | 0.001 |
| September | 0.3865 | 0.001 |
| October | 0.3367 | 0.001 |
| November | 0.3950 | 0.001 |
| December | 0.5292 | 0.001 |

**Table S3.** Set of the best (lowest AIC) generalized mixed model selection for annual reported cases. The model selected is highlighted in bold.

| Annual temporal correlation* | Annual population density* | Annual cattle density* | Annual pig density* | Elevation* | Slope* | Primary rice crop arable area* | Primary rice yield* | Ssecondary rice crop arable area* | df | LL** | AIC | ΔAIC |
| --- | --- | --- | --- | --- | --- | --- | --- | --- | --- | --- | --- | --- |
| 0.0705 | 0.2342 | - | - | 0.6434 | - | 0.6662 | - | - | 11 | -1936.78 | 3895.57 | 0.00 |
| - | **0.2396** | **-** | **-** | **0.6554** | **-** | **0.6717** | **-** | **-** | **10** | **-1937.89** | **3895.78** | **0.21** |
| 0.0703 | 0.2143 | - | -0.0876 | 0.6350 | - | 0.6643 | - | - | 12 | -1936.34 | 3896.68 | 1.11 |
| - | 0.2193 | - | -0.0896 | 0.6468 | - | 0.6697 | - | - | 11 | -1937.44 | 3896.88 | 1.31 |
| 0.0694 | 0.2418 | 0.0423 | - | 0.6453 | - | 0.6546 | - | - | 12 | -1936.68 | 3897.36 | 1.80 |
| - | 0.2483 | 0.0497 | - | 0.6574 | - | 0.6579 | - | - | 11 | -1937.75 | 3897.50 | 1.93 |
| 0.0704 | 0.2343 | - | - | 0.6497 | -0.0164 | 0.6658 | - | - | 12 | -1936.77 | 3897.54 | 1.98 |
| 0.0713 | 0.2349 | - | - | 0.6467 | - | 0.6605 | - | 0.0076 | 12 | -1936.78 | 3897.55 | 1.99 |
| 0.0707 | 0.2344 | - | - | 0.6407 | - | 0.6665 | -0.0104 | - | 12 | -1936.78 | 3897.56 | 2.00 |

* coefficient

**LL=Log likelihood

**Table S4.** Set of the best (lowest AIC) generalized mixed model selection for monthly reported cases. The model selected is highlighted in bold.

| Monthly temporal correlation* | Average pig density* | Average buffalo density* | Monthly soil moisture* | Monthly temperature range* | Monthly percentage of NDVI-2* | df | LL** | AIC | ΔAIC |
| --- | --- | --- | --- | --- | --- | --- | --- | --- | --- |
| 0.1180 | **-** | **-** | **0.2781** | **-0.2397** | **-0.0478** | **11** | **-2597.24** | **5216.48** | **0** |
| 0.1181 | -0.0853 | - | 0.2772 | -0.2400 | -0.0475 | 12 | -2596.97 | 5217.93 | 1.45 |
| 0.1180 | - | 0.0748 | 0.2779 | -0.2398 | -0.0477 | 12 | -2597.13 | 5218.26 | 1.78 |
| 0.1181 | -0.0960 | 0.0943 | 0.2769 | -0.2402 | -0.0474 | 13 | -2596.79 | 5219.59 | 3.10 |
| 0.1195 | - | - | 0.2445 | -0.2596 | - | 10 | -2600.41 | 5220.83 | 4.34 |

* coefficient

**LL=Log likelihood

**2. Supplementary Results for percentage of flooded area**

**Table S5.** Set of the best (lowest AIC) generalized mixed model selection for monthly reported cases with the percentage of flooded area variable instead of the temperature range. The model selected is highlighted in bold.

| Monthly temporal correlation* | Average pig density* | Average buffalo density* | Monthly soil moisture* | Monthly percentage of flooded area * | Monthly percentage of NDVI-2* | df | LL** | AIC | ΔAIC |
| --- | --- | --- | --- | --- | --- | --- | --- | --- | --- |
| 0.1287 | **-** | **-** | **0.2708** | **0.2475** | **-** | **10** | **-2601.20** | **5222.41** | **0** |
| 0.1287 | -0.0792 | - | 0.2703 | 0.2474 | - | 11 | -2600.97 | 5223.94 | 1.54 |
| 0.1287 | - | 0.0804 | 0.2706 | 0.2476 | - | 11 | -2601.08 | 5224.16 | 1.75 |
| 0.1285 | - | - | 0.2736 | 0.2451 | -0.0032 | 11 | -2601.19 | 5224.39 | 1.98 |
| 0.1288 | -0.0907 | 0.0993 | 0.2699 | 0.2476 | - | 12 | -2600.79 | 5225.57 | 3.16 |

* coefficient

**LL=Log likelihood

**Table S6.** Results of the final generalized linear mixed model for monthly reported cases

| Variables | OR (95% Confidence Interval) | P-value |
| --- | --- | --- |
| Monthly temporal correlation | 1.1373 (1.0951-1.1811) | <0.0001 |
| Monthly soil moisture | 1.3110 (1.2223-1.4062) | <0.0001 |
| Monthly percentage of flooded area | 1.2808 (1.2170-1.3479) | <0.0001 |

*OR=Odds ratio

**
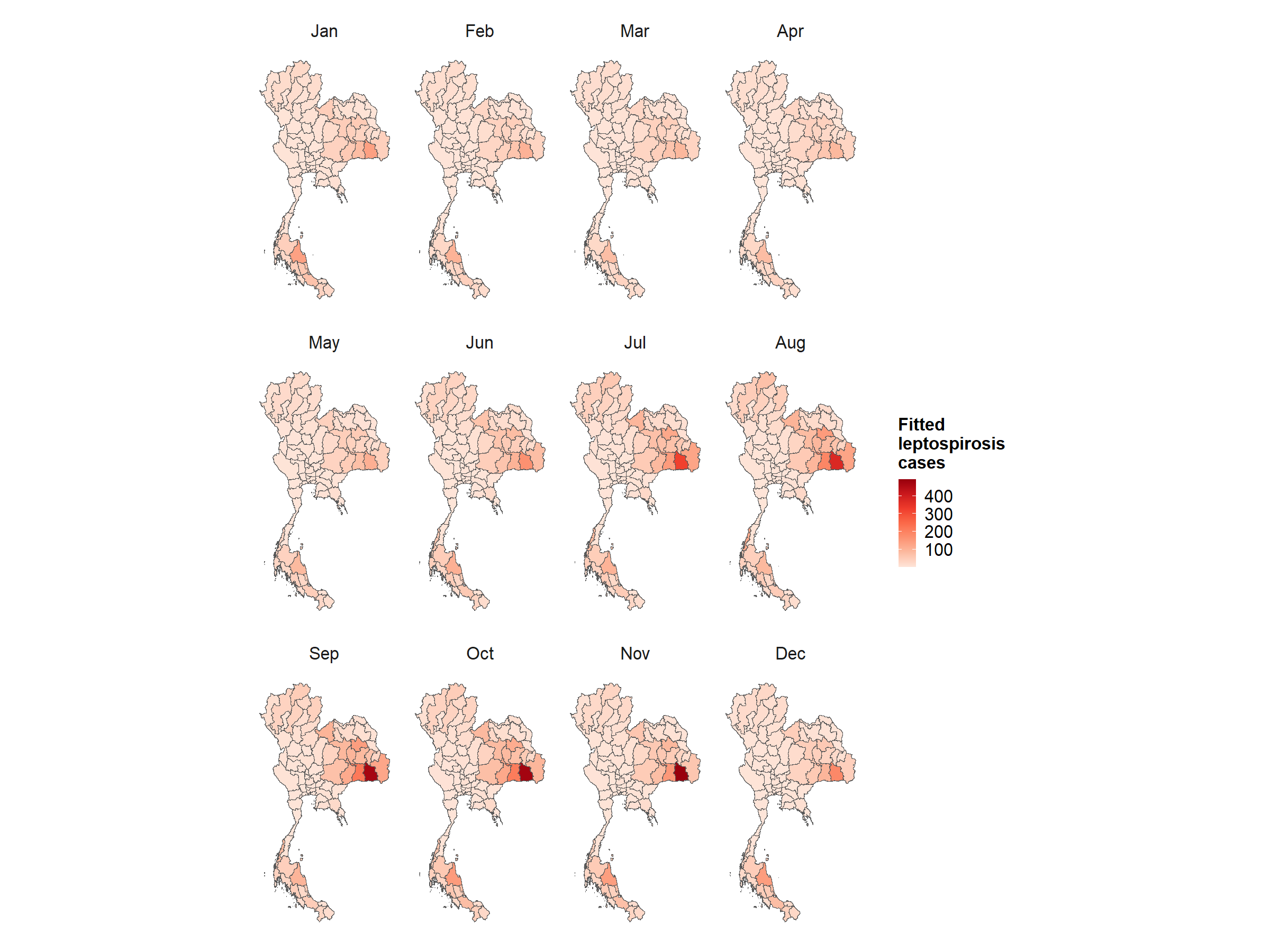
**

**Fig. S5.** Plots of monthly leptospirosis cases estimated by the best GLMM for percentage of flooded area. Maps created using R Program version 4.0.3 (https://www.r-project.org/).

**
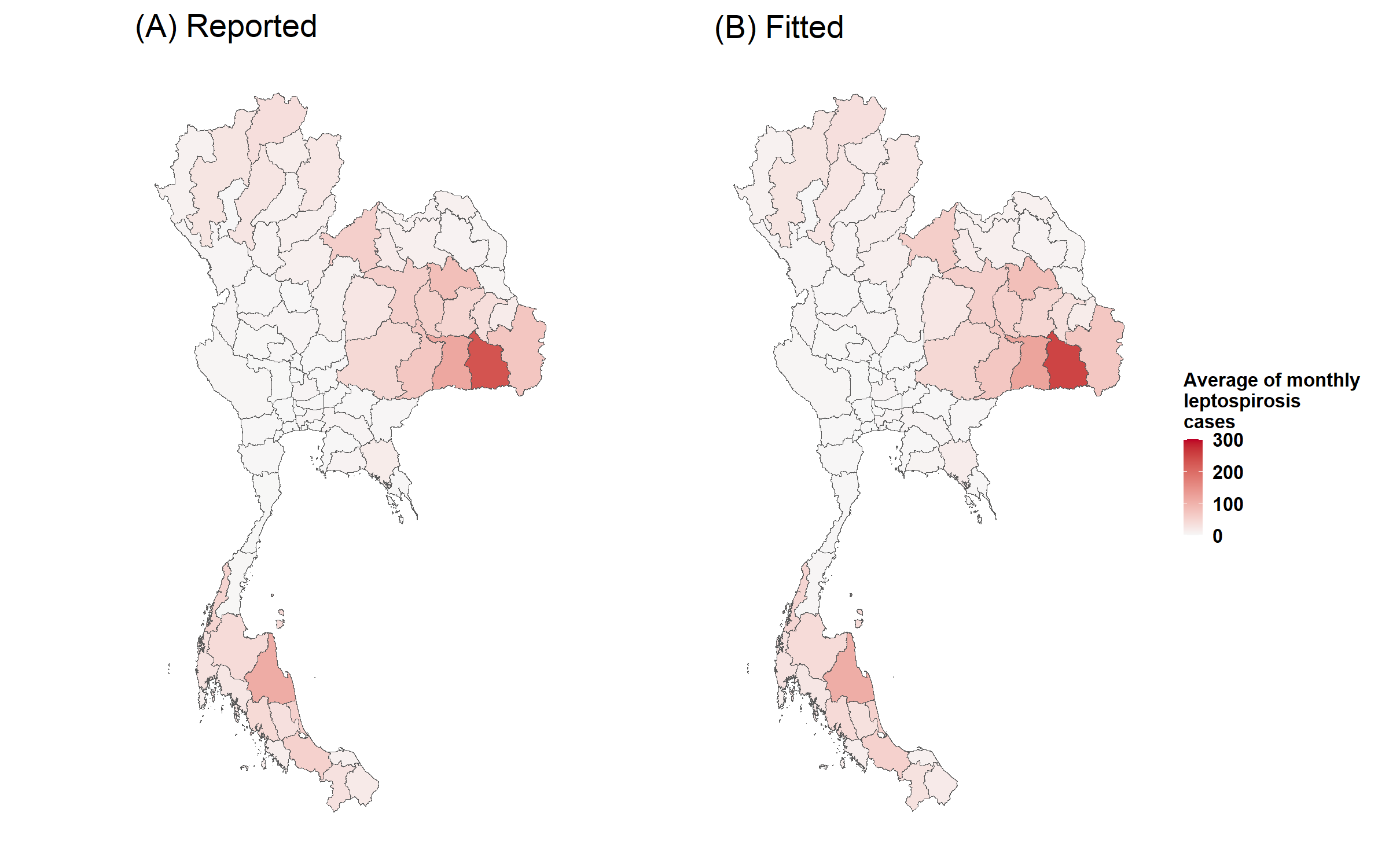
**

**Fig. S6.** Plots of comparison between average monthly reported leptospirosis cases (A) and average monthly fitted leptospirosis cases estimated by the best GLMM for percentage of flooded area. Maps created using R Program version 4.0.3 (https://www.r-project.org/).

**3. Supplementary Data**


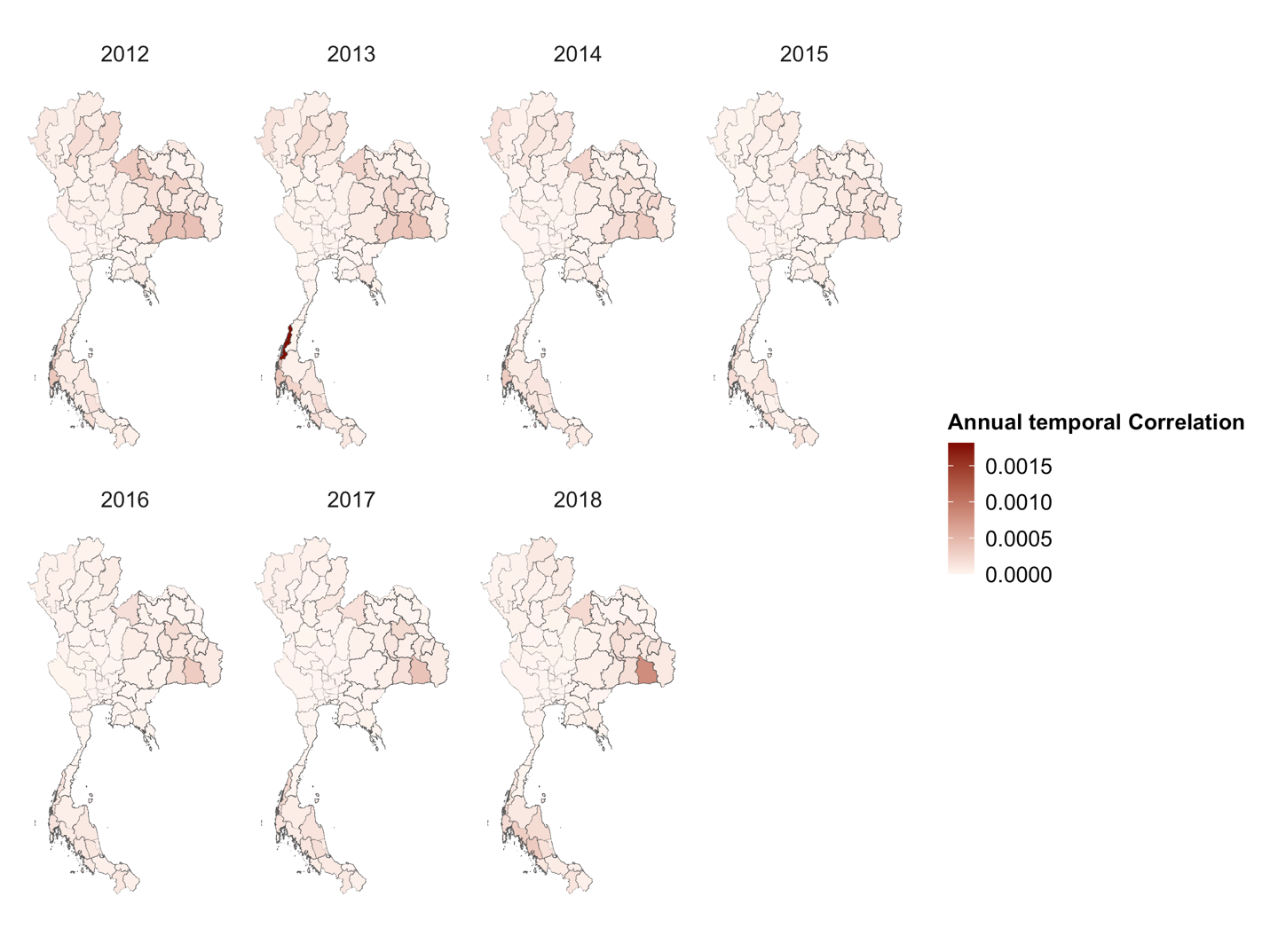


**Fig. S7.** Annual temporal correlation, Thailand 2012–2018. Maps created using R Program version 4.0.3 (https://www.r-project.org/).


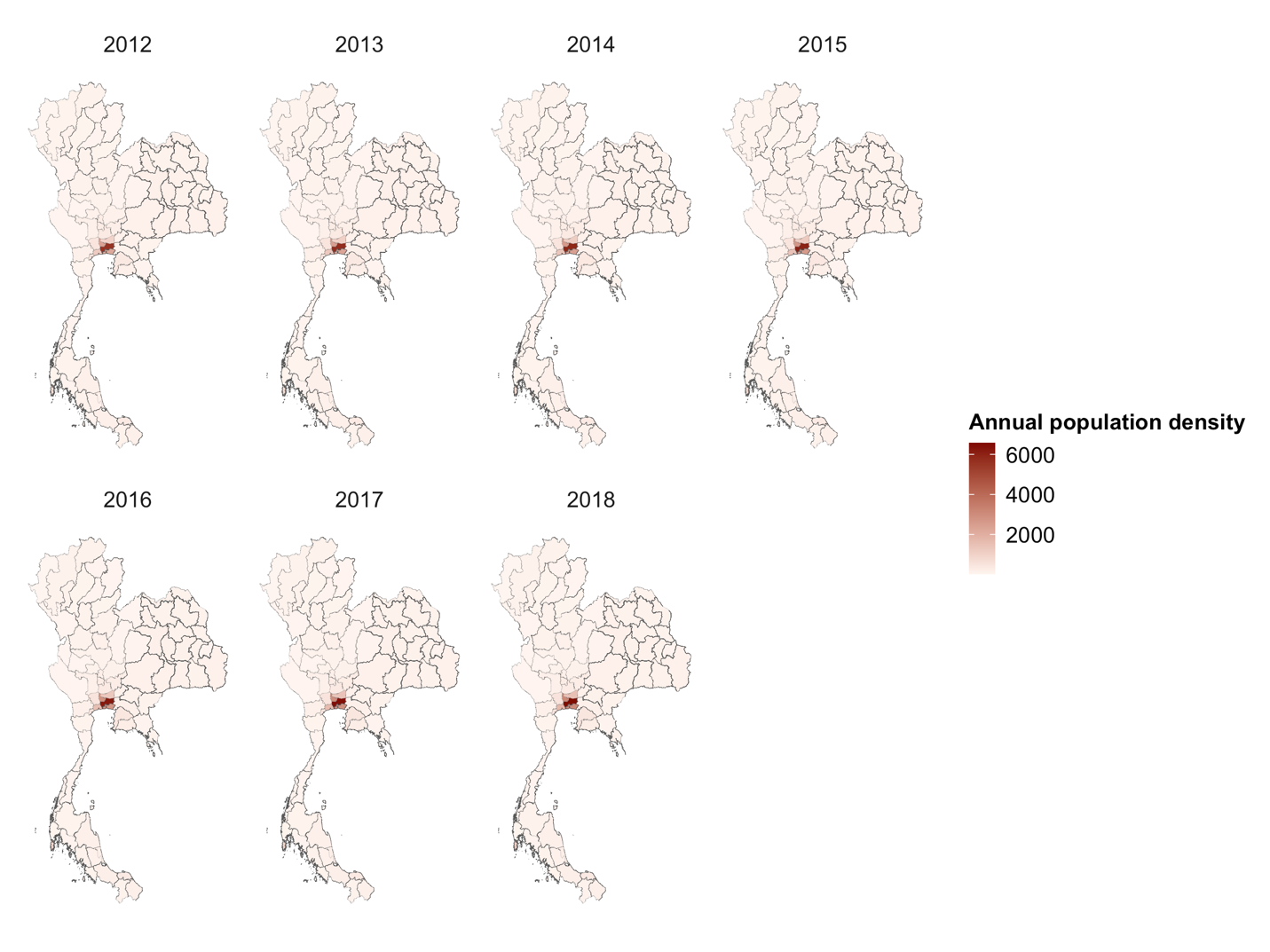


**Fig. S8.** Annual population density, Thailand 2012–2018. Maps created using R Program version 4.0.3 (https://www.r-project.org/).


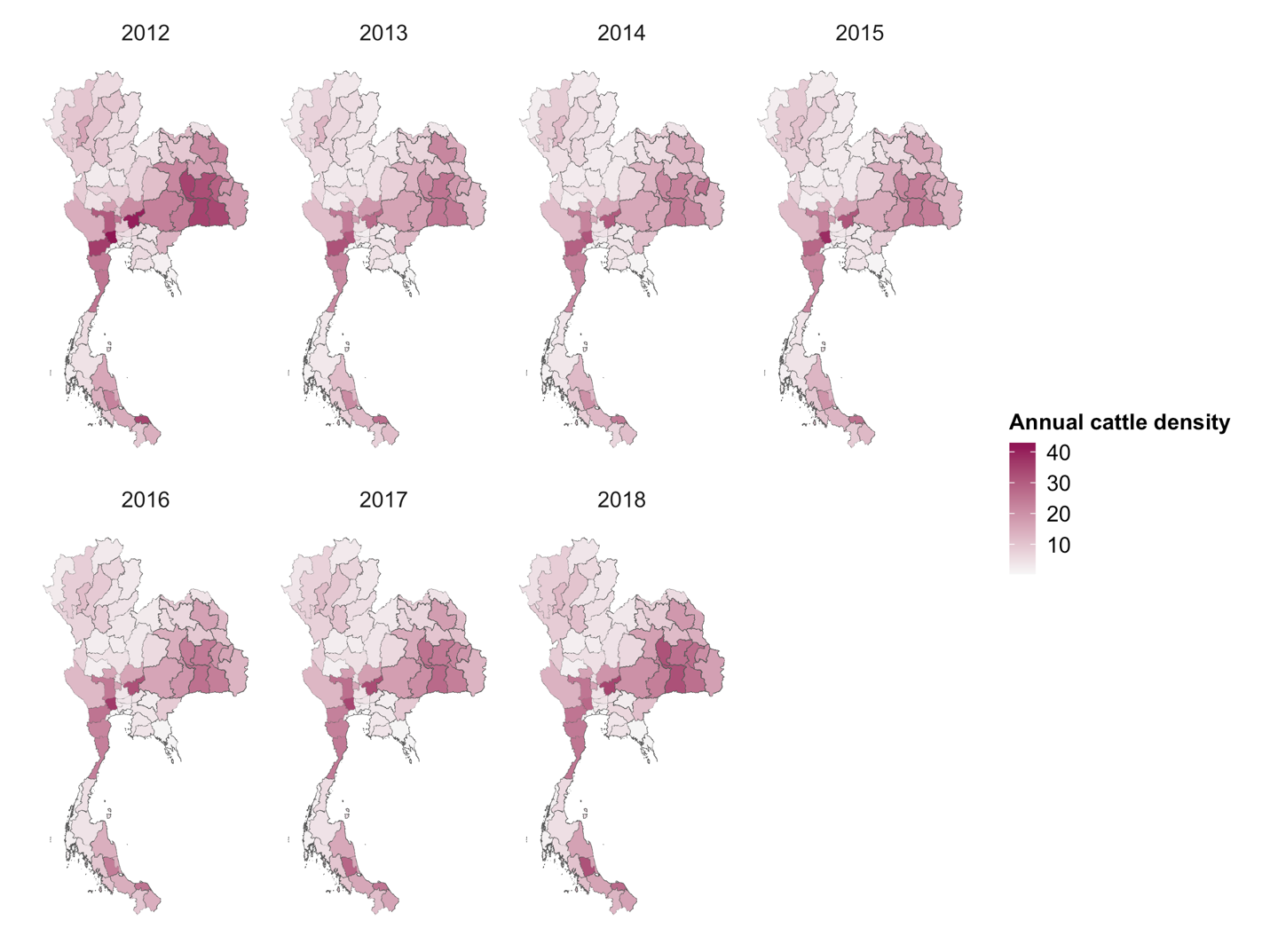


**Fig. S9.** Annual cattle density, Thailand 2012–2018. Maps created using R Program version 4.0.3 (https://www.r-project.org/).


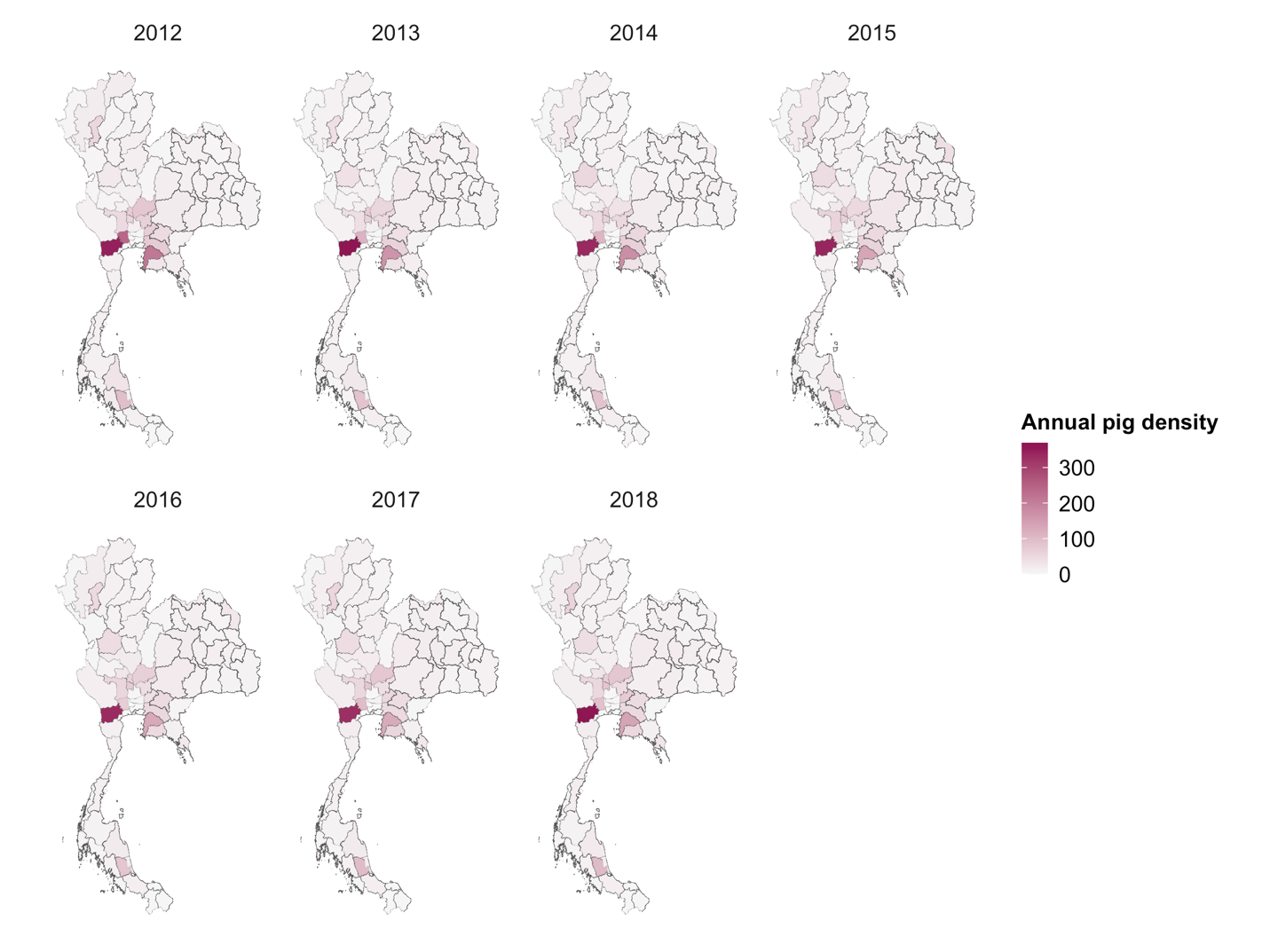


**Fig. S10.** Annual pig density, Thailand 2012–2018. Maps created using R Program version 4.0.3 (https://www.r-project.org/).


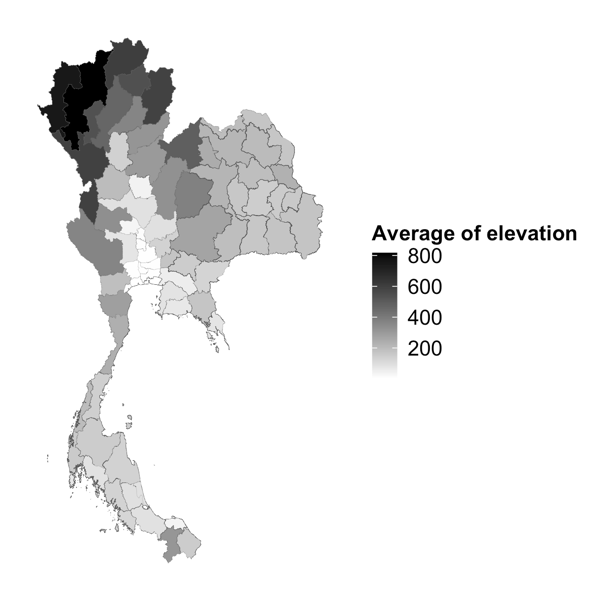


**Fig. S11.** Average of elevation, Thailand 2012–2018. Maps created using R Program version 4.0.3 (https://www.r-project.org/).


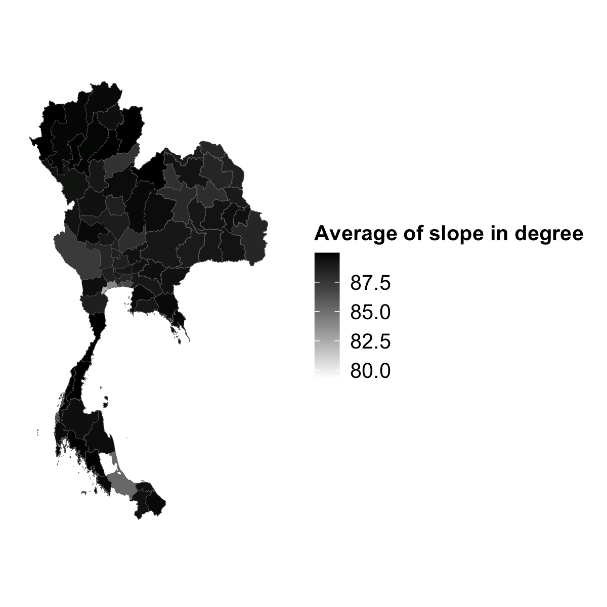


**Fig. S12.** Average of slope in degree, Thailand 2012–2018. Maps created using R Program version 4.0.3 (https://www.r-project.org/).


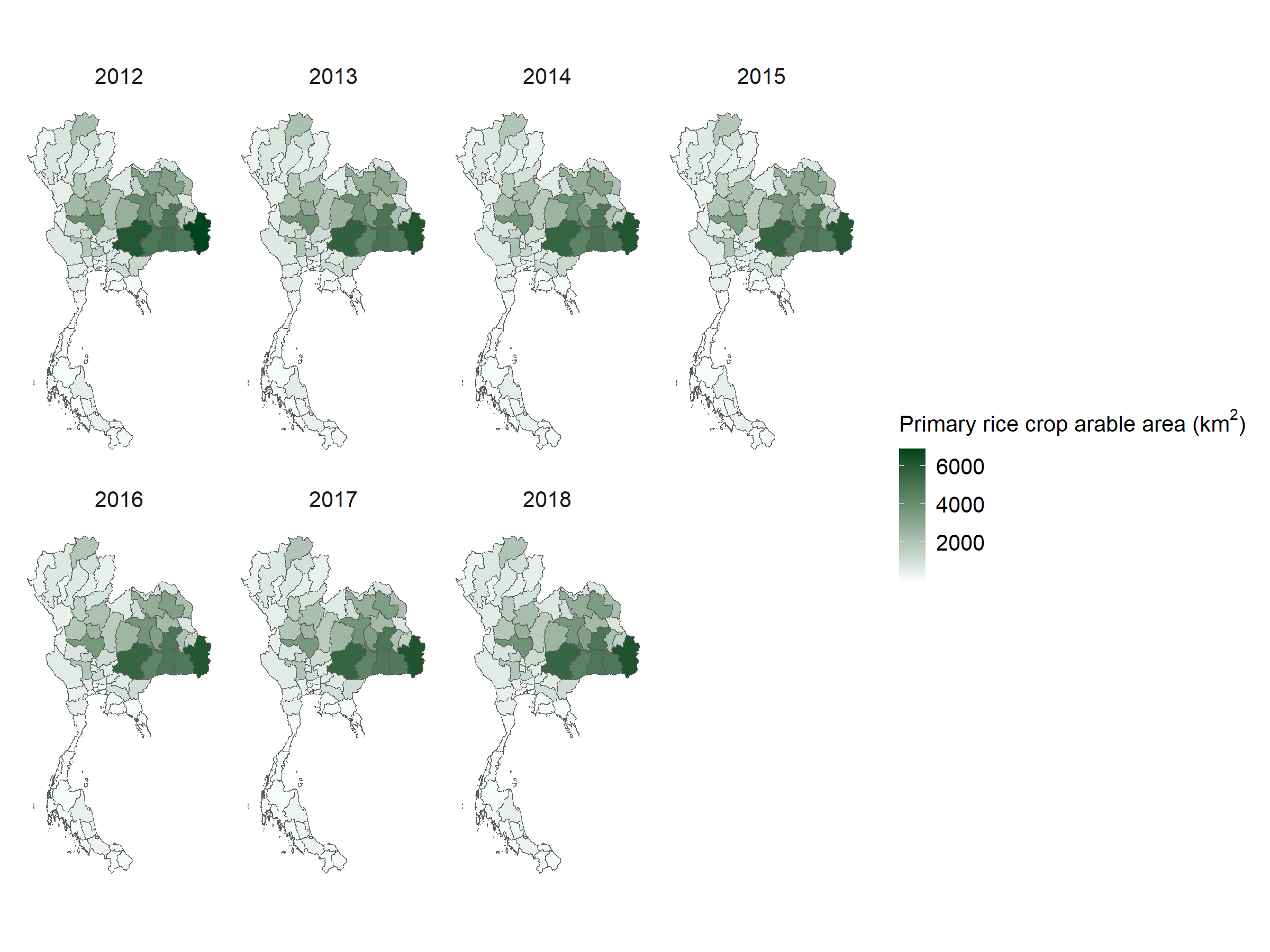


**Fig. S13.** Primary rice crop arable area, Thailand 2012–2018. Maps created using R Program version 4.0.3 (https://www.r-project.org/).


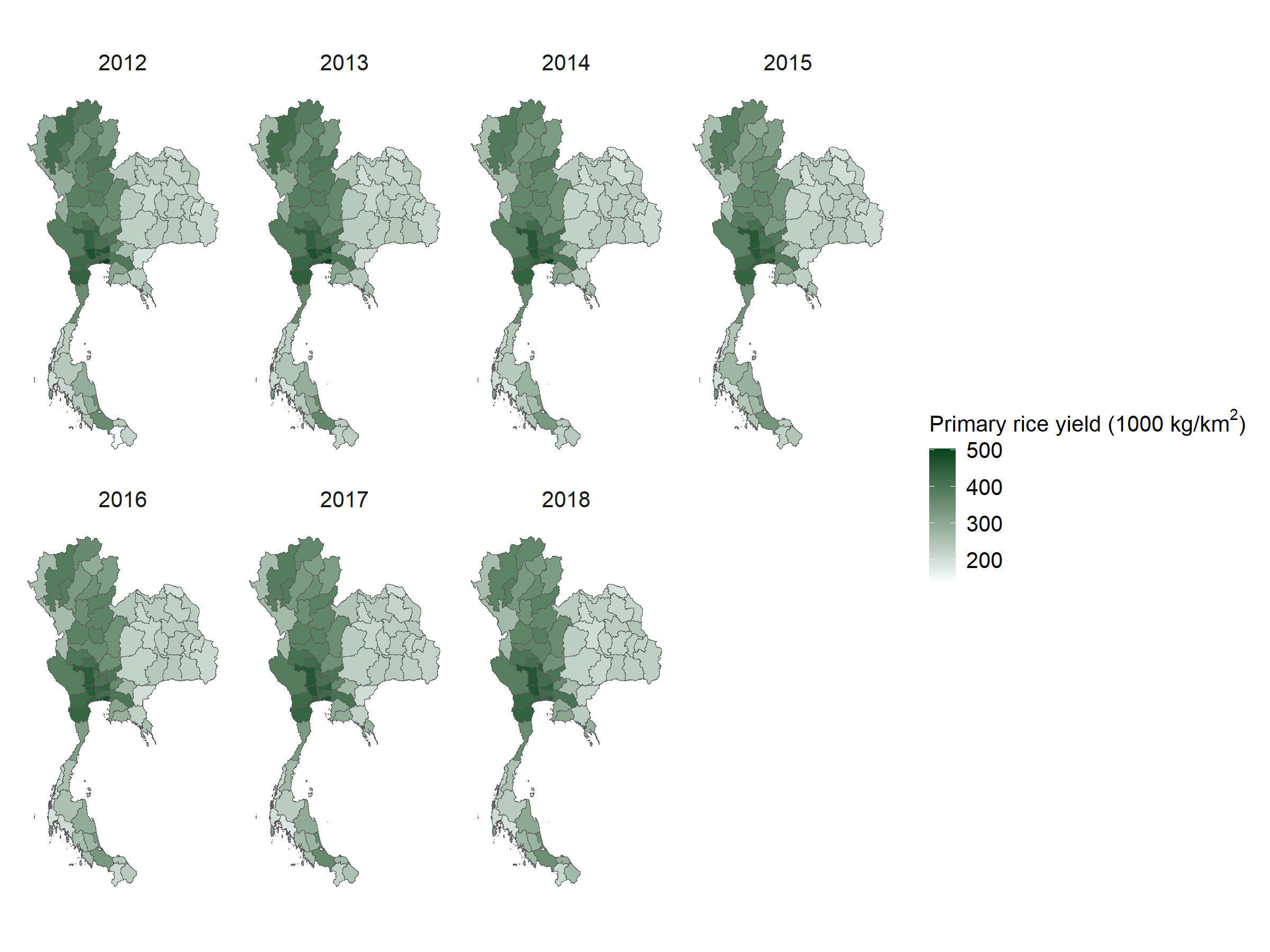


**Fig. S14.** Primary rice yield, Thailand 2012–2018. Maps created using R Program version 4.0.3 (https://www.r-project.org/).


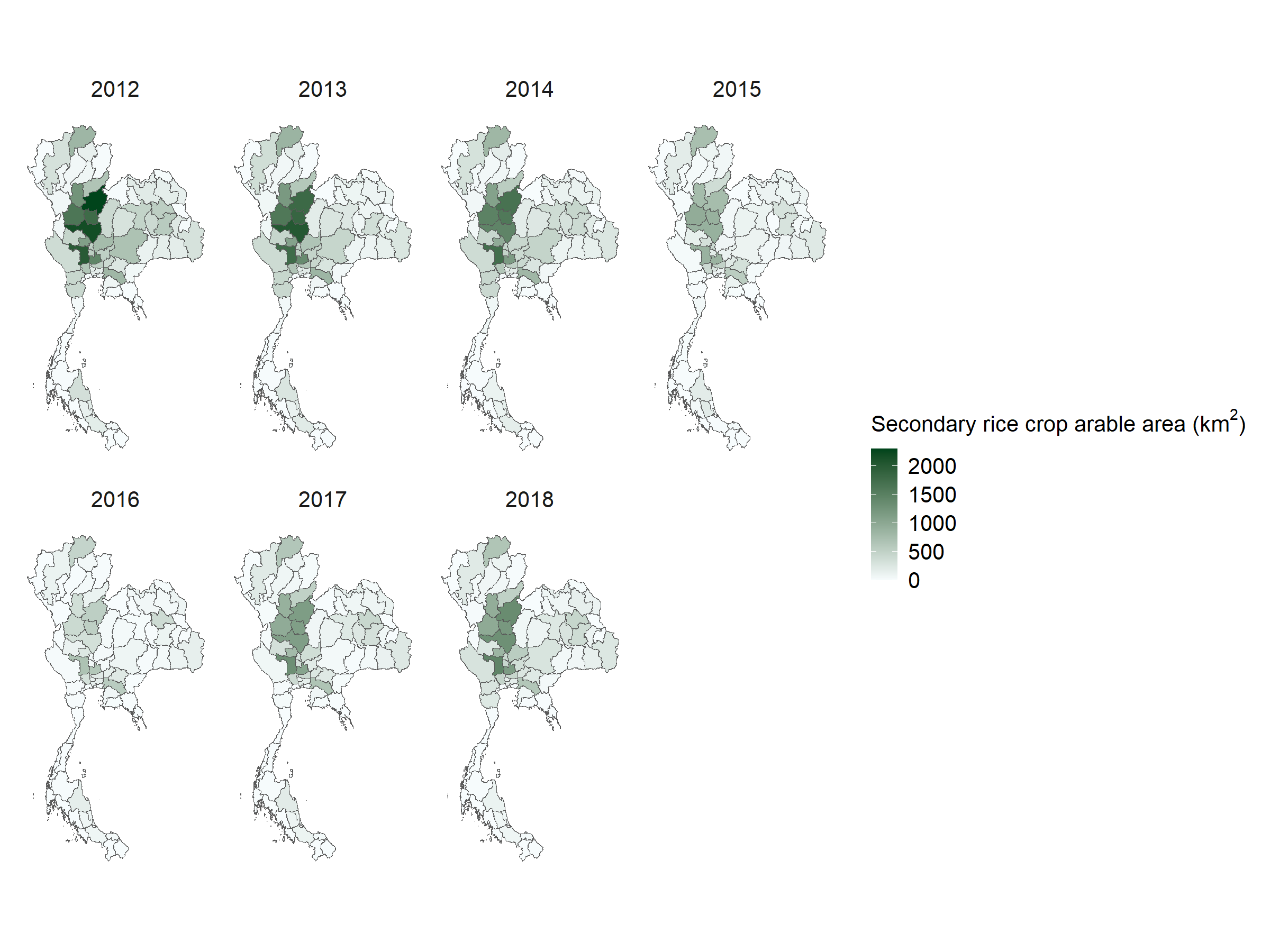


**Fig. S15.** Secondary rice crop arable area, Thailand 2012–2018. Maps created using R Program version 4.0.3 (https://www.r-project.org/).


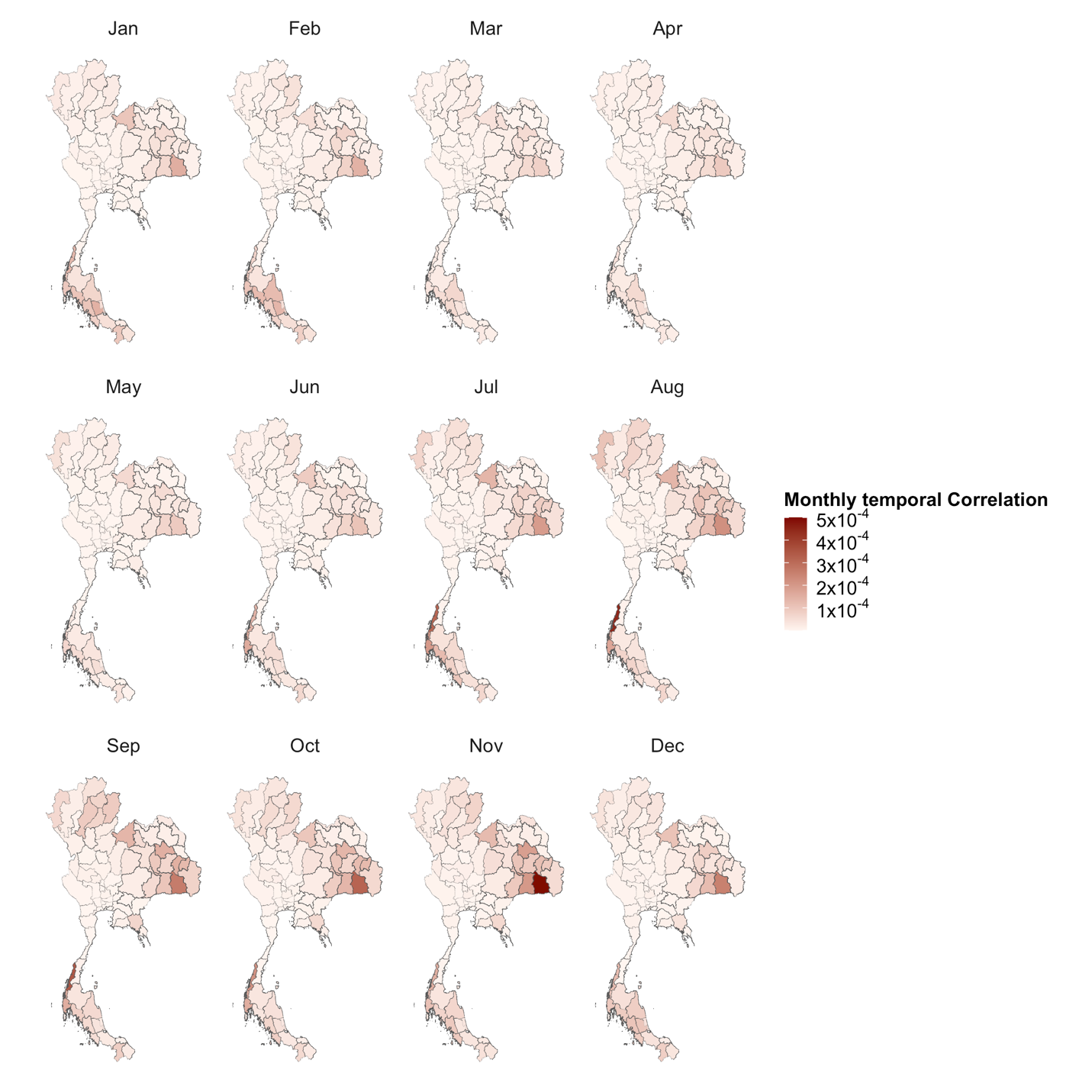


**Fig. S16.** Monthly temporal correlation, Thailand. Maps created using R Program version 4.0.3 (https://www.r-project.org/).


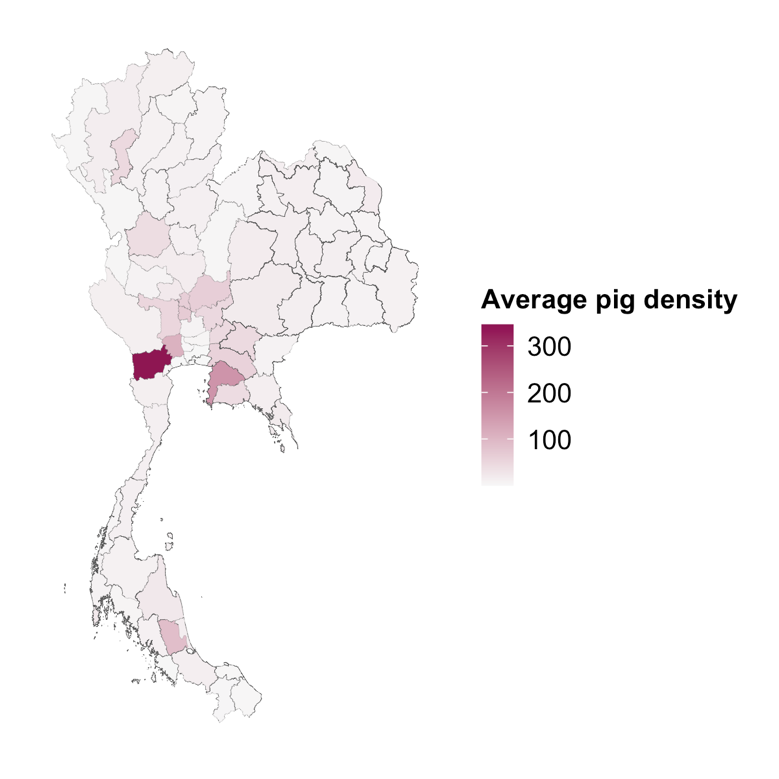


**Fig. S17.** Average pig density, Thailand for all years. Maps created using R Program version 4.0.3 (https://www.r-project.org/).


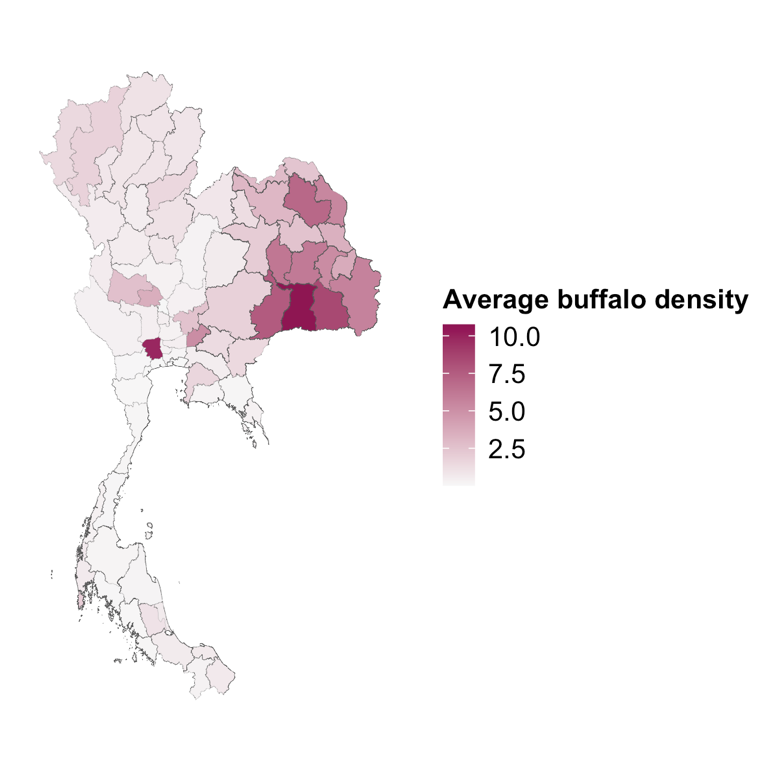


**Fig. S18.** Average buffalo density, Thailand for all years. Maps created using R Program version 4.0.3 (https://www.r-project.org/).


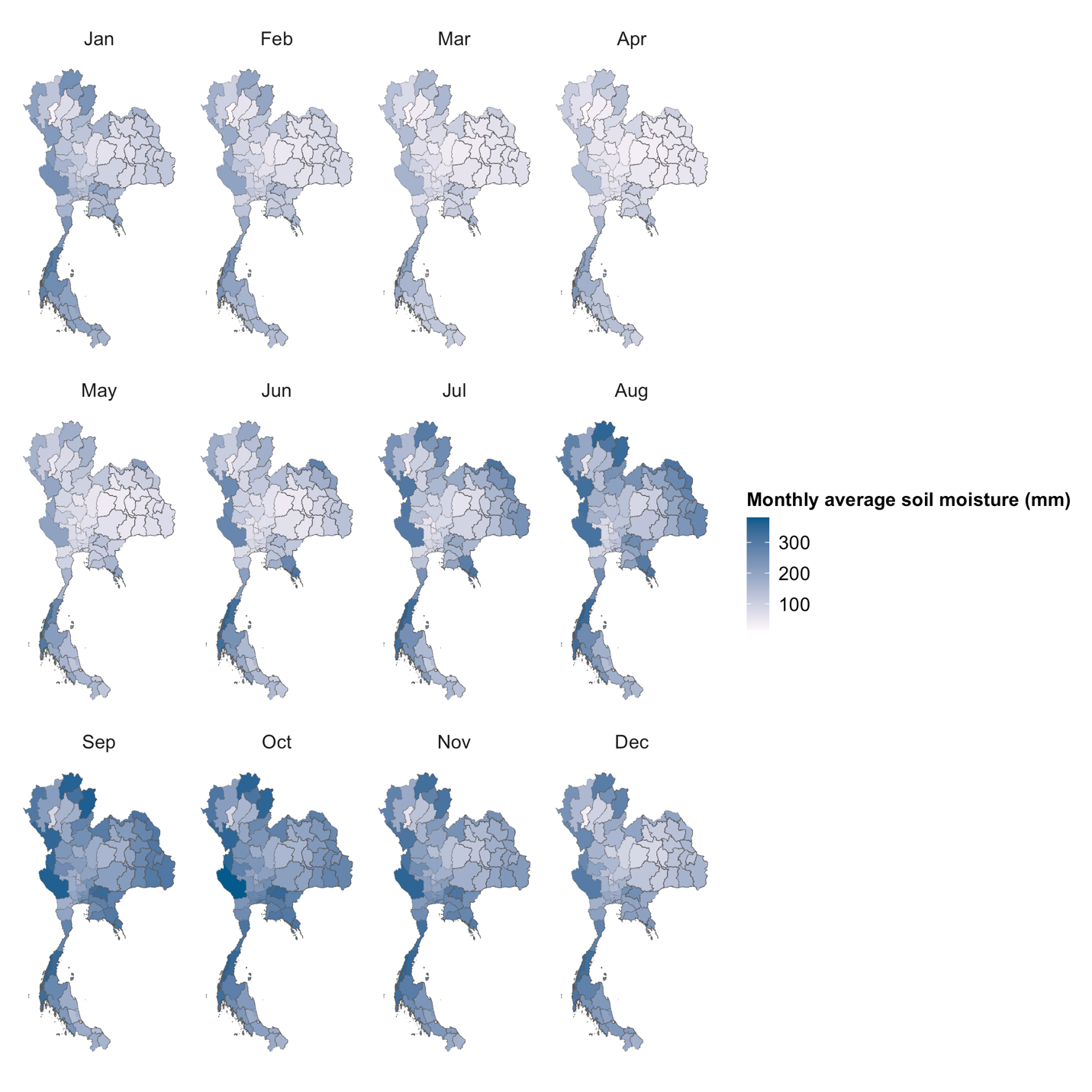


**Fig. S19.** Monthly average soil moisture, Thailand. Maps created using R Program version 4.0.3 (https://www.r-project.org/).


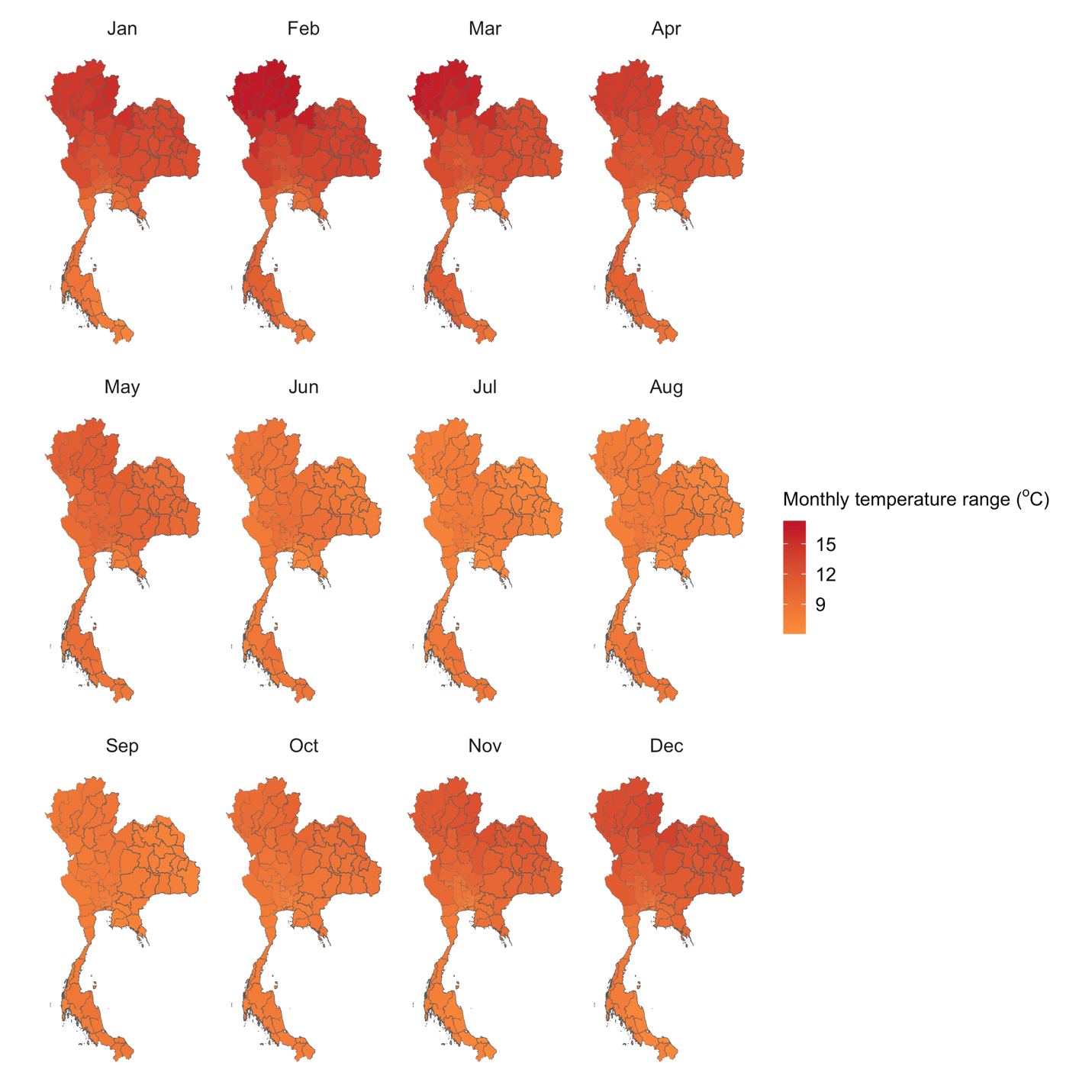


**Fig. S20.** Monthly temperature range, Thailand. Maps created using R Program version 4.0.3 (https://www.r-project.org/).


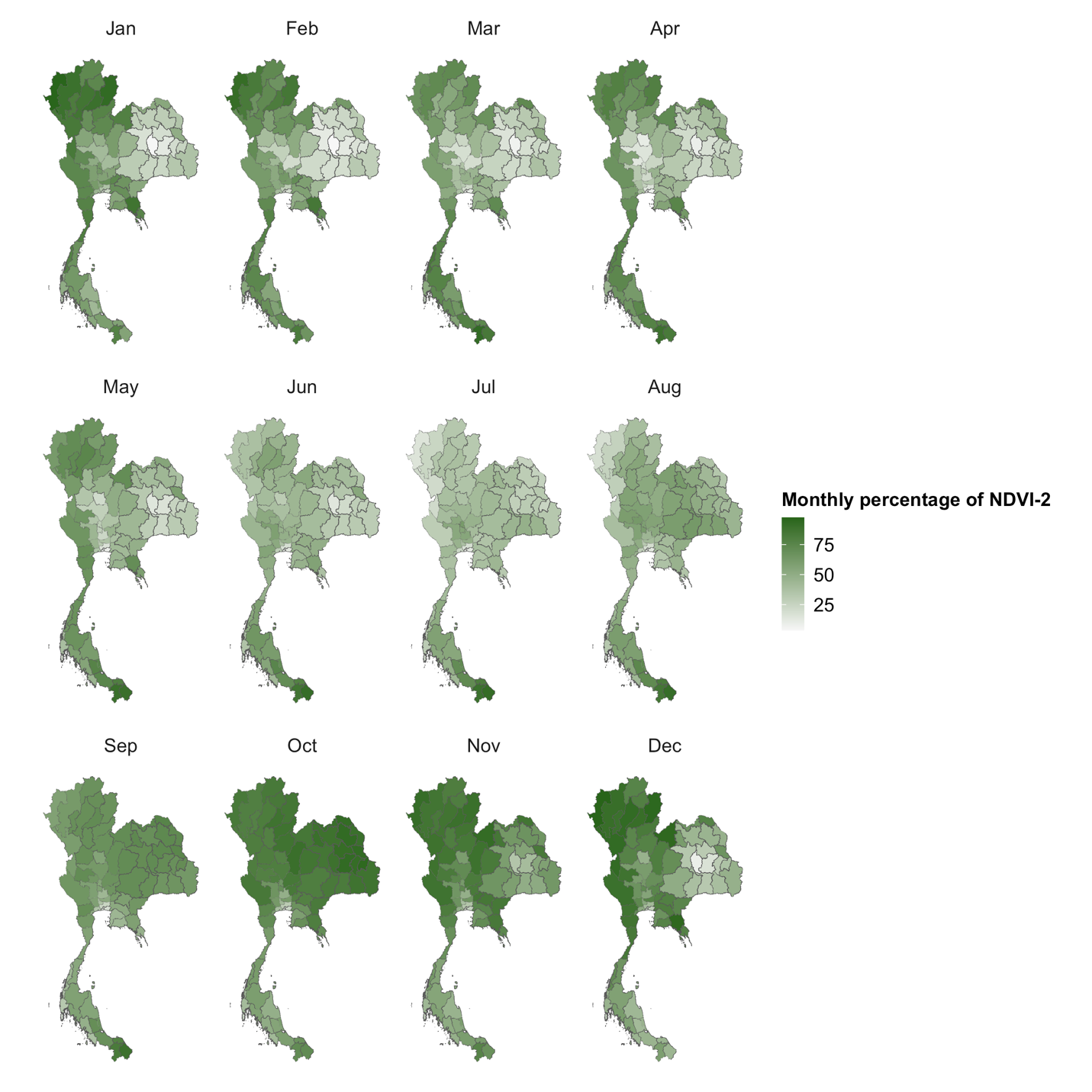


**Fig. S21.** Monthly percentage of NDVI-2 area, Thailand. Maps created using R Program version 4.0.3 (https://www.r-project.org/).


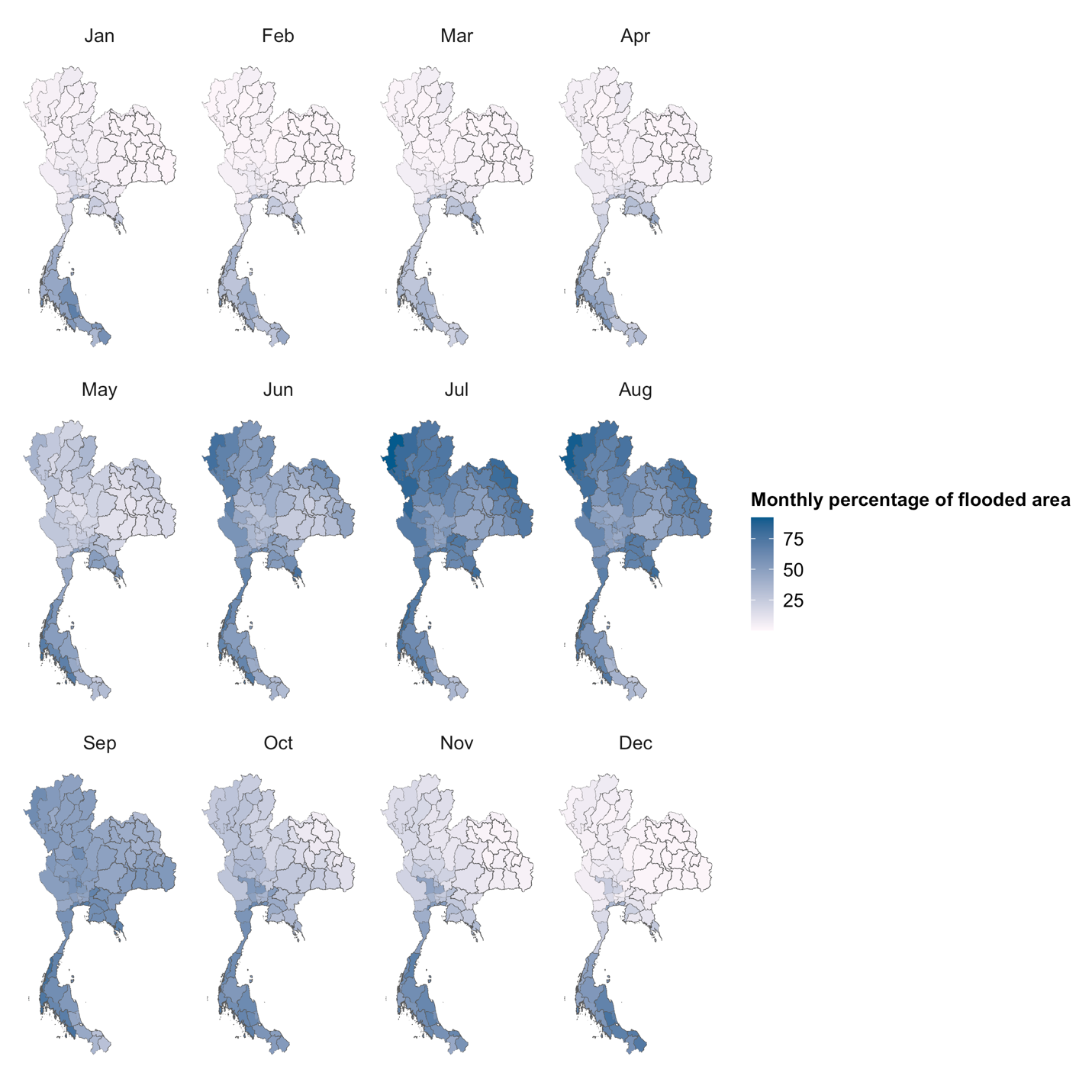


**Fig. S22.** Monthly percentage of flooded area, Thailand. Maps created using R Program version 4.0.3 (https://www.r-project.org/).
